# Molecular Evolution and Functional Divergence of Trace Amine–Associated Receptors

**DOI:** 10.1371/journal.pone.0151023

**Published:** 2016-03-10

**Authors:** Seong-il Eyun, Hideaki Moriyama, Federico G. Hoffmann, Etsuko N. Moriyama

**Affiliations:** 1 School of Biological Sciences, University of Nebraska-Lincoln, Lincoln, NE, 68588, United States of America; 2 Center for Biotechnology, University of Nebraska-Lincoln, Lincoln, NE, 68588, United States of America; 3 Department of Biochemistry, Molecular Biology, Entomology, and Plant Pathology and Institute for Genomics, Biocomputing, and Biotechnology, Mississippi State University, Mississippi State, MS, 39762, United States of America; 4 Center for Plant Science Innovation, University of Nebraska-Lincoln, Lincoln, NE, 68588, United States of America; Scripps Health and The Scripps Research Institute, UNITED STATES

## Abstract

Trace amine-associated receptors (TAARs) are a member of the G-protein-coupled receptor superfamily and are known to be expressed in olfactory sensory neurons. A limited number of molecular evolutionary studies have been done for TAARs so far. To elucidate how lineage-specific evolution contributed to their functional divergence, we examined 30 metazoan genomes. In total, 493 TAAR gene candidates (including 84 pseudogenes) were identified from 26 vertebrate genomes. TAARs were not identified from non-vertebrate genomes. An ancestral-type TAAR-like gene appeared to have emerged in lamprey. We found four therian-specific TAAR subfamilies (one eutherian-specific and three metatherian-specific) in addition to previously known nine subfamilies. Many species-specific TAAR gene duplications and losses contributed to a large variation of TAAR gene numbers among mammals, ranging from 0 in dolphin to 26 in flying fox. TAARs are classified into two groups based on binding preferences for primary or tertiary amines as well as their sequence similarities. Primary amine-detecting TAARs (TAAR1-4) have emerged earlier, generally have single-copy orthologs (very few duplication or loss), and have evolved under strong functional constraints. In contrast, tertiary amine-detecting TAARs (TAAR5-9) have emerged more recently and the majority of them experienced higher rates of gene duplications. Protein members that belong to the tertiary amine-detecting TAAR group also showed the patterns of positive selection especially in the area surrounding the ligand-binding pocket, which could have affected ligand-binding activities and specificities. Expansions of the tertiary amine-detecting TAAR gene family may have played important roles in terrestrial adaptations of therian mammals. Molecular evolution of the TAAR gene family appears to be governed by a complex, species-specific, interplay between environmental and evolutionary factors.

## Introduction

Biogenic amines, such as histamine, serotonin, adrenaline, and dopamine, are enzymatic decarboxylation products of amino acids. They are crucial intercellular signaling molecules that function widely as neurotransmitters and neuromodulators [1, 2]. In addition to these classical amines, there is another class of endogenous amines, called ‘‘trace amines” (TAs), that are present in mammalian tissues at trace amounts (0.1–10 nM) [3–5]. They include 2-phenylethylamine (PEA), m-tyramine, ρ-tyramine, *meta*-octopamine, *para*-octopamine, 3-iodothyronamine, tryptamine, and *N*,*N*-dimethyltryptamine. Trace amine-associated receptors (TAARs) were originally identified based on their relatedness to biogenic amine receptors and discovered in search of the receptors activated by the TAs in the brain [6, 7]. Liberles and Buck [8] demonstrated later that TAARs also function as chemosensory receptors expressed in the olfactory epithelium in mouse. TAAR4, for example, is stimulated by PEA, which is a carnivore odor that evokes physiological and behavioral responses in two prey species (rat and mouse) [9]. Deletion of TAAR4 in mice was also shown to specifically eliminate the high-sensitivity responses to PEA and puma urine volatiles [10]. TAARs thus play important roles in sensing predator and prey odors. Recently in zebrafish, TAAR13c has been shown to give high-affinity responses to cadaverine (1,5-diaminopentane, a major product of fish tissue decay) and related aliphatic diamines with odd chains of medium length [11]. Ferrero *et al*. [12] furthermore showed that TAARs can be classified into two groups (TAAR1-4 *vs*. TAAR5-9) based on whether they preferentially detect primary or tertiary amines (RNH_2_ or R_3_N).

While there are other types of biogenic amine receptors such as serotonin-gated cation channel in vertebrates and biogenic amine-gated chloride channels in invertebrates [2], TAARs and almost all biogenic amine receptors belong to the G-protein-coupled receptor (GPCR) superfamily. GPCRs are characterized by seven hydrophobic transmembrane regions with three intracellular and three extracellular loops. They mediate signal transduction in response to a wide variety of stimuli and represent the largest multi-gene family in animal genomes. For example, there are more than 900 GPCRs in human [13] and more than 1,800 in mouse [14]. Within the GPCR superfamily, TAARs as well as biogenic amine receptors belong to the Class A: Rhodopsin-like family [7]. In the mouse genome, for example, fifteen functional genes and one pseudogene are known for TAARs. They are classified into nine subfamilies (TAAR1 through TAAR9). In mouse, most of these subfamilies are represented by single copy genes except for TAAR7, which includes five genes and one pseudogene, and TAAR8, which includes three genes [15]. All mouse TAARs except for TAAR1 are expressed in the main olfactory epithelium (MOE) [8, 16]. TAAR1 is expressed in the brain [7]. The olfactory receptors (ORs) in mammals, another Class A family of GPCRs, also are predominantly expressed in the MOE [17]. The sensory neurons in the mammalian MOE thus have two types of chemosensory receptors, TAARs and ORs.

In vertebrates, the comparative genomics and molecular evolution of the OR family has been studied extensively in teleost fishes and tetrapods [18–20]. Taken together, these studies show that while the tetrapod genomes have a large number of OR genes, ranging from 400 to 2,100, a significant portion of them, in the order of 20–50%, are pseudogenes [20]. Although the OR genes are scattered over almost all chromosomes [21, 22], they are mainly generated by tandem gene duplications [23]. Species-specific expansions of OR genes are found in several species and these expansions have been linked to ecological adaptations [24]. A recent study of the *Nematostella vectensis* (sea anemone) genome indicated that the origin of vertebrate ORs can be traced back to the Cnidaria [25].

Unlike the OR family, a limited number of molecular evolutionary studies have been done for TAARs. The complete TAAR gene set has been described in nine mammalian species (human, chimpanzee, macaque, mouse, rat, dog, cow, opossum, and platypus) [15, 26, 27], chicken [28], six teleosts (fugu, spotted green pufferfish, stickleback, medaka, zebrafish, and Atlantic salmon), a cartilaginous fish (elephant shark), and a jawless fish (sea lamprey) [26, 29, 30]. These studies showed that the tetrapod genomes have small numbers of TAAR genes (3–22 genes), while many teleost fishes have higher numbers of TAAR genes compared to tetrapods, ranging from 13 to 109 genes.

The goal of this study is to understand the molecular evolutionary process of the TAAR gene family. We focused on elucidating how species-specific duplication contributed to their functional divergence among mammals. We identified complete repertoires of TAAR genes and pseudogenes from 30 metazoan genomes, especially from 17 species of mammals. We found that the size of the TAAR family varies significantly among mammals. While the largest number of TAARs, 26 functional genes, was found in the flying fox genome, no functional TAAR genes were found in the dolphin genome. In addition to the previously known nine subfamilies, we identified four subfamilies all therian-specific (found only in marsupials and placental mammals). Among the mammalian-specific TAAR subfamilies, TAAR7 was found to be subject to rapid species-specific gene duplications in many species. We also found that TAARs have two different evolutionary patterns. Primary amine-detecting TAARs (TAAR1-4) appear to be evolving under strong negative selection, whereas tertiary amine-detecting TAARs (TAAR5-9) have significant variations in gene numbers and many of them appear to evolve under the influence of positive selection, reflecting complex species-specific relationships between environmental and evolutionary factors.

## Results and Discussion

### Identification of TAAR Genes

Using previously reported TAAR protein sequences as queries, we searched TAAR candidates from 30 metazoan genomes (S1 Table). A total of 493 TAAR genes (including 84 pseudogenes) were identified from 26 vertebrate genomes (Table 1; see S2 Table for the details). Our analyses failed to identify TAAR candidates in any of the four non-vertebrate genomes we examined (an amphioxus, two tunicates, and a sea anemone). Gnathostome (jawed vertebrate) paralogs were classified based on sequence similarities and on phylogenetic analyses. The nine TAAR subfamilies (TAAR1-9) were easily recognized from the tetrapod genomes. We also identified four new mammalian-specific subfamilies (E1 and M1-M3) (described later). Consistent with the previous findings [26], one group of TAAR-like genes was found only in teleosts (zebrafish, stickleback, medaka, and spotted green pufferfish) and a frog; they were designated as the TAAR subfamily V (TAAR V) following Hashiguchi and Nishida [26]. In our search using the TAAR V profile hidden Markov model, we confirmed that TAAR V was found only in the genomes of two teleost fishes (fugu, *Takifugu rubripes*, and spotted green pufferfish, *Tetraodon nigroviridis*) and a frog (*Xenopus tropicalis*) but not in any other tetrapod species we examined.

**Table 1:** The number of TAAR genes identified in the 30 animal genomes.

Group/Species name	Common name	Totalnumber^a^	Number of TAAR subfamily genes^b^																	
			T1	T2	T3	T4	T5	T6	T7	T8	T9	TE1	TM1	TM2	TM3	TFI	TFII	TFIII	TL	TV
**[Euarchontoglires]**																				
*Homo sapiens*	human	6 (3)	1	1	0 (1)	0 (1)	1	1	0 (1)	1	1	0	0	0	0	0	0	0	0	0
*Mus musculus*	house mouse	15 (1)	1	1	1	1	1	1	5 (1)	3	1	0	0	0	0	0	0	0	0	0
*Rattus norvegicus*	Norway rat	17 (2)	1	1	1	1	1	1	7 (2)	3	1	0	0	0	0	0	0	0	0	0
**[Laurasiatheria]**																				
*Bos taurus*	cow	21 (8)	1	1^c^	1	1	1	5 (2)	7 (4)	3 (2)	1	0	0	0	0	0	0	0	0	0
*Tursiops truncatus*	bottlenosed dolphin	0 (3)	0 (2)	0	0	0	0	0	0	0	0 (1)	0	0	0	0	0	0	0	0	0
*Equus caballus*	horse	11 (4)	1	1	1	1	2	1	1 (1)	2 (1)	1 (1)	0 (1)	0	0	0	0	0	0	0	0
*Canis familiaris*	dog	2 (2)	0 (1)	0 (1)	0	1	1	0	0	0	0	0	0	0	0	0	0	0	0	0
*Pteropus vampyrus*	Malayan flying fox	26 (10)	1	1^c^	0 (1)	1	1	4 (6)	16	1 (3)	1	0	0	0	0	0	0	0	0	0
*Myotis lucifugus*	little brown bat	6 (1)	1	1^c^	1	1	1	0 (1)	0	0	1	0	0	0	0	0	0	0	0	0
*Sorex araneus*	common shrew	9 [1] (3)	1	[1] (1)	1	1 (1)	1	3	2	0	0 (1)	2	0	0	0	0	0	0	0	0
*Erinaceus europaeus*	hedgehog	6 [2] (4)	[1] (1)	1^c^	1	0 (2)	0 (1)	[1]	2	0	1	1	0	0	0	0	0	0	0	0
**[Afrotheria]**																				
*Echinops telfairi*	lesser hedgehog tenrec	9 [1] (7)	1 (1)	1^c^ (2)	1	1	0	0 (1)	2 (1)	1	[1]	2 (2)	0	0	0	0	0	0	0	0
*Loxodonta africana*	African elephant	9 [3] (3)	[1]	1^c^	[1]	1 [1]	1	1	2	2 (3)	0	1	0	0	0	0	0	0	0	0
**[Xenarthra]**																				
*Dasypus novemcinctus*	nine-banded armadillo	5 (4)	1	1^c^	1	0	1	0	1 (1)	0 (2)	0 (1)	0	0	0	0	0	0	0	0	0
**[Marsupialia]**																				
*Macropus eugenii*	tammar wallaby	18 [1] (3)	(1)	[1]	1	1	1	0	0	0	1	0	1 (1)	9 (1)	4	0	0	0	0	0
*Monodelphis domestica*	opossum	22 (4)	1	1^c^	1	3 (1)	1	0	0	0	7 (1)	0	1	2 (1)	5 (1)	0	0	0	0	0
**[Prototheria]**																				
*Ornithorhynchus anatinus*	platypus	4 (1)	1	1^c^	1	1 (1)	0	0	0	0	0	0	0	0	0	0	0	0	0	0
**[Sauropsida]**																				
*Gallus gallus*	chicken	4 (1)	1	2^c^	0	0	1 (1)	0	0	0	0	0	0	0	0	0	0	0	0	0
*Taeniopygia guttata*	zebra finch	1 (0)	1	0	0	0	0	0	0	0	0	0	0	0	0	0	0	0	0	0
*Anolis carolinensis*	Carolina anole	3 (0)	1	1^c^	0	0	1	0	0	0	0	0	0	0	0	0	0	0	0	0
**[Amphibia]**																				
*Xenopus tropicalis*	pipid frog	7 (0)	1	0	0	5	0	0	0	0	0	0	0	0	0	0	0	0	0	1
**[Teleostei]**																				
*Takifugu rubripes*	fugu (Japanese pufferfish)	18 (1)	0	0	0	0	0	0	0	0	0	0	0	0	0	14 (1)	0	3	0	1
*Tetraodon nigroviridis*	spotted green pufferfish	34 (3)	0	0	0	0	0	0	0	0	0	0	0	0	0	12 (3)	0	21	0	1
*Danio rerio*	zebrafish	110 (10)^d^	1	0	0	0	0	0	0	0	0	0	0	0	0	92 (8)	11	6 (2)	0	1
**[Chondrichthyes]**																				
*Callorhinchus milii*	elephant shark	2 (3)	1 (1)	0	0	1 (2)^e^	0	0	0	0	0	0	0	0	0	0	0	0	0	0
**[Agnatha]**																				
*Petromyzon marinus*	sea lamprey	25 (3)	0	0	0	0	0	0	0	0	0	0	0	0	0	0	0	0	25 (3)	0
**[Cephalochordata]**																				
*Branchiostoma floridae*	amphioxus	0																		
**[Urochordata]**																				
*Ciona intestinalis*	vase tunicate	0																		
*Ciona savignyi*	tunicate	0																		
**[Cnidaria]**																				
*Nematostella vectensis*	sea anemone	0																		

^a^TAAR gene candidates are divided into three categories: intact, incomplete, and pseudogenes. The first number shown is that of "intact" genes, which contain full-length open reading frames with seven complete transmembrane regions. The number of "incomplete" genes due to incomplete genome sequences (*e*.*g*., long ambiguous sequences such as a run of N’s or contig ends) or incompletely identified exons (*e*.*g*., TAAR2, see below) is given in square brackets. The number in parentheses is that of possible pseudogenes, which contain premature stop codons or frame-shifting insertions or deletions.

^b^T1-T9, TE1, TM1-TM3, TFI-III, TL, and TV indicate TAAR1-9, TAAR E1, TAAR M1-M3, fish-specific TAAR I-III, lamprey TAAR-like genes, and TAAR V, respectively. The group names of fish-specific TAAR I-III and TAAR V are given by Hashiguchi and Nishida [26].

^c^Only the exon2 sequences (coding 304 to 331 amino acids) were identified from these TAAR2 genes. The exon1 (coding 8 to 20 amino acids) can be located more than 6000 bp upstream.

^d^The sequences are from Hashiguchi and Nishida [26]. We classified them into five subfamilies.

^e^These three shark sequences (S2a, S2bP, and S2cP) are most similar to TAAR4. However, as we described, these shark TAARs may have diverged from the ancestral TAARs before the divergence of TAAR2-4 (see also phylogenies in Figs 2 and 3, and S1 Fig).

### Synteny of TAAR Loci among Tetrapod Species

**Fig 1:**
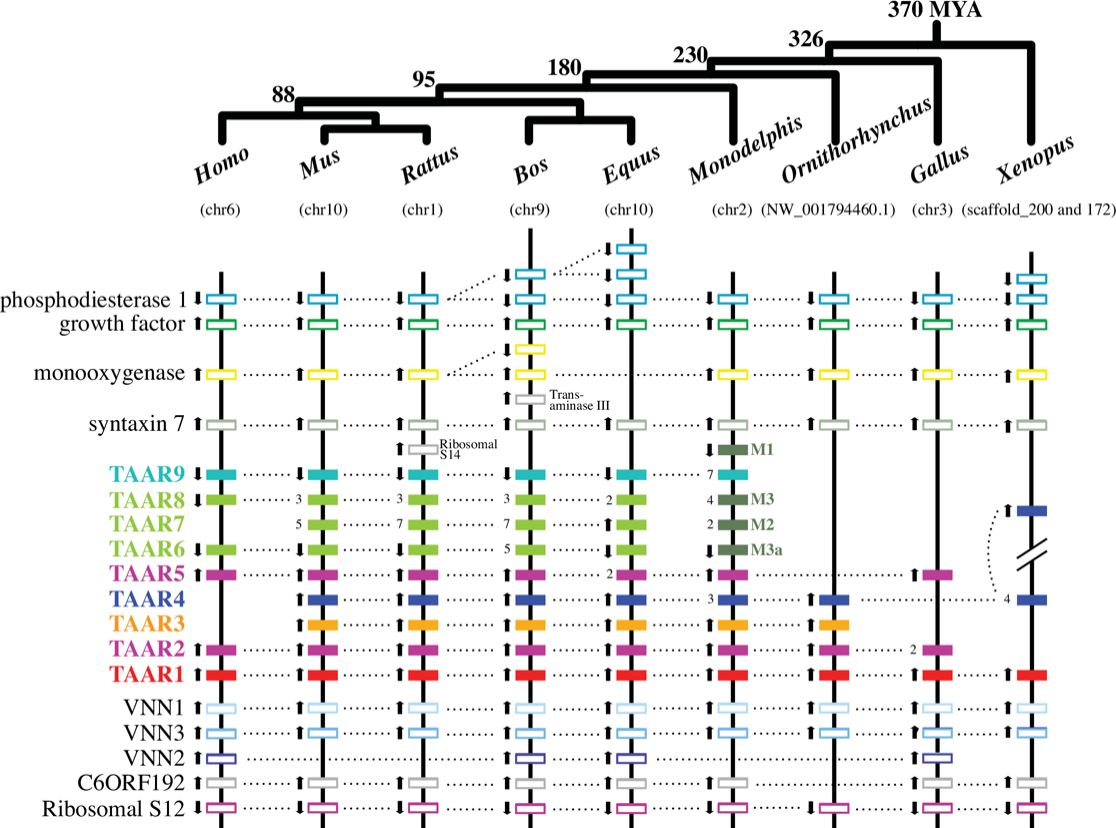
Syntenic relationship of the TAAR genes in nine vertebrate genomes. Only genomes in which all TAAR genes are located in one chromosome or no more than two scaffolds were examined (*Xenopus* genes are found in two scaffolds). TAAR and adjacent non-TAAR genes are depicted by the closed and open boxes, respectively (pseudogenes are not included). TAAR genes are shown in different colors based on their taxonomic distributions as follows: TAAR1 found in jawed vertebrates in red, amniote-specific TAAR2 and TAAR5 in purple, mammalian-specific TAAR3 in orange, tetrapod-specific TAAR4 in dark blue, eutherian-specific TAAR6, 7, and 8 in light green, therian-specific TAAR9 in cyan, and metatherian-specific TAARM1-M3 in dark green. Note that the same color scheme is used in Figs 2 and 3. When tandemly duplicated functional copies exist for a TAAR gene, the copy number is also shown. Gene locations are not in scale (see S2 Table for the actual positional information). Black arrows indicate transcriptional directions. A current consensus of the tetrapod phylogeny with their approximate divergence times (million years ago; MYA) is illustrated at the top [33, 34]. The chromosome or scaffold numbers are shown below the genus names.

TAAR genes in human, mouse, opossum, and chicken are known to be located on a single chromosome, while teleost TAARs are scattered over multiple chromosomes [15, 26]. We analyzed the distribution of the TAAR and other adjacent genes in nine representative tetrapods and summarized the results in Fig 1. The syntenic relationships of TAARs and the adjacent genes are highly conserved as a single gene cluster. At least in amniotic genomes (mammals and chicken), the TAAR genes are all clustered in the specific region of a single chromosome. The average length of intergenic regions between two adjacent TAARs is 12,235 bps for five eutherian species (human, mouse, rat, cow, and horse). The transcriptional orientations are highly consistent among orthologs (Fig 1). We observed many tandem duplications especially in TAAR6, TAAR7, and TAAR8, which are all eutherian specific. All tetrapod TAAR genes we examined are nested between Vanin (VNN) and Syntaxin 7 (STX7) genes. VNN1 is associated with pantetheinase activity [31]. STX7 protein forms a SNARE complex and is involved in protein-trafficking [32]. It is not known, however, if these adjacent genes and TAARs are co-expressed or share functional relationships.

### Origin and Early Evolution of TAARs

**Fig 2:**
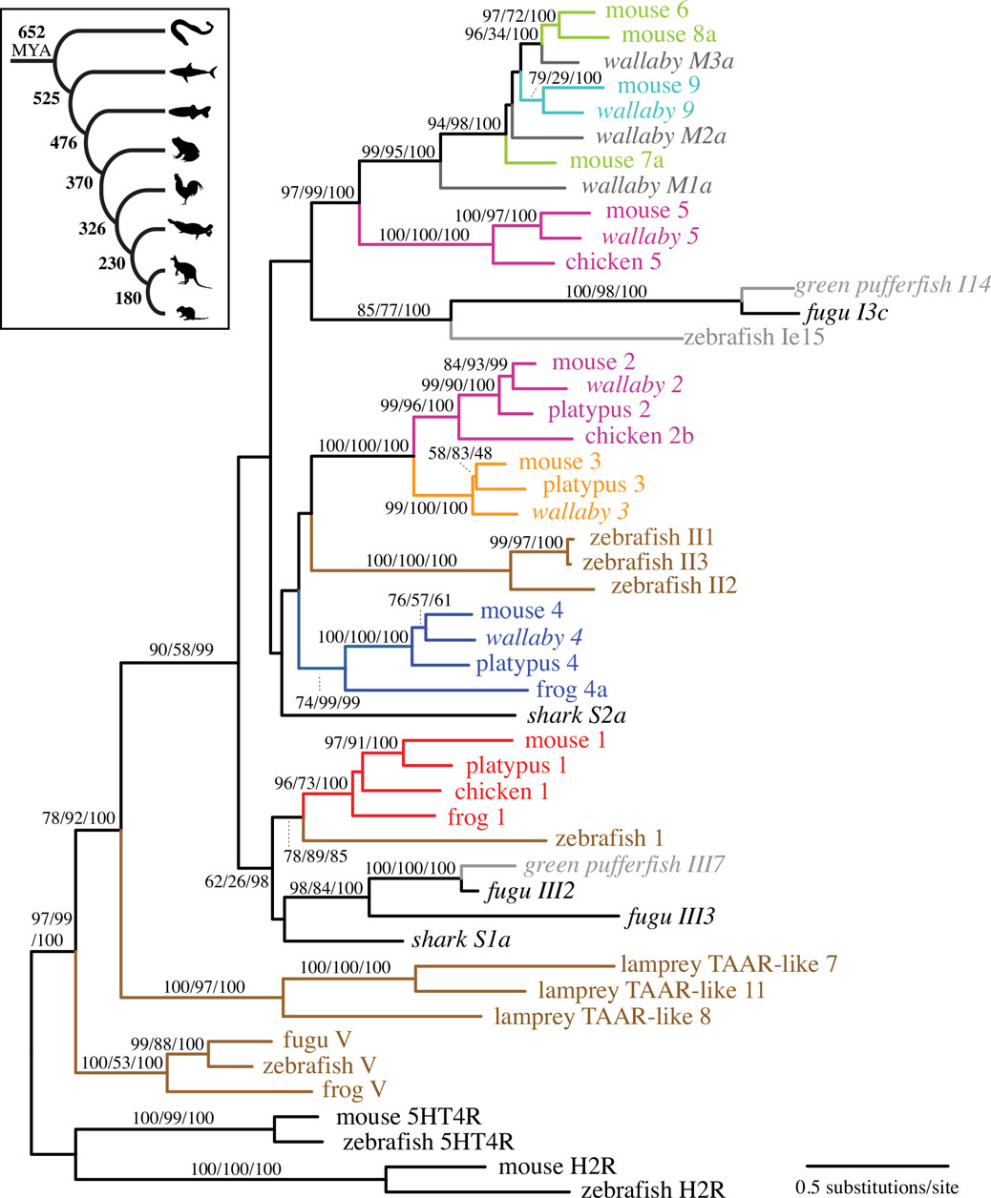
The maximum-likelihood phylogeny of TAAR proteins from ten representative vertebrate species. Only representative TAAR proteins are included for each species. Four biogenic amine receptors (5HT4R: serotonin receptors, and H2R: histamine receptors) are used as the outgroup. The genes newly identified in this study are shown in italics. The numbers at internal branches show the bootstrap support values (%) for the maximum-likelihood and neighbor-joining phylogenies and the posterior probability (%) for the Bayesian phylogeny in this order. Supporting values are shown only for the internal branches that have at least one method supporting higher than 70%. For TAAR V, teleost TAARs, and lamprey TAAR-like, we followed the gene names given by Hashiguchi and Nishida [26]. The inset illustrates a current consensus of the vertebrate phylogeny with their approximate divergence times (MYA) [33, 34].

Fig 2 shows the phylogeny of the representative TAAR proteins from five tetrapods (mouse, tammar wallaby, platypus, chicken, and frog), three teleosts (fugu, spotted green pufferfish, and zebrafish), a cartilaginous fish (elephant shark), and a jawless fish (sea lamprey). This phylogeny clusters TAAR subfamilies into three strongly supported monophyletic groups: TAAR V, lamprey TAAR-like, and TAARs found in gnathostomes (jawed vertebrates) (TAAR1-9). The TAAR V group is clearly clustered separately from other GPCRs including biogenic amine receptors, and located most basal after these GPCRs (Fig 2 and S1 Fig). This family seems to have been maintained only in teleost and amphibian lineages but lost from other vertebrates. All 25 TAAR-like proteins found from the sea lamprey (*Petromyzon marinus*) genome form a well-supported monophyletic group (100% bootstrap value, S1 Fig). Our phylogenetic analysis indicates that the sea lamprey TAAR-like genes and the gnathostome TAAR1-9 shared the direct common ancestor. While Hashiguchi and Nishida [26] showed the TAAR V genes to be more closely related to gnathostome TAARs, their phylogeny showed no significant support for the location of TAAR V and lamprey TAAR-like groups. Furthermore, as described in Materials and Methods, the TAAR signature motif (S2 Fig) was only weakly conserved both in TAAR V and in the sea lamprey TAAR-like genes, but was present in the majority of the gnathostome members of the TAAR subfamilies. These results suggest that the well-conserved TAAR motif, and also the TAAR function, appeared after jawed vertebrates (gnathostomes) diverged from jawless fish, about 652 million years ago (MYA) [33].

Cartilaginous fish represent one of the earliest branches of the gnathostome tree (see the inset of Fig 2). The elephant shark (*Callorhinchus milii*), the representative of this group in our study, possesses two distinct TAAR genes in its genome: TAAR S1a and TAAR S2a. These two elephant shark TAARs maintain the TAAR signature motif (S3 Fig). Ortholog relationship between the shark TAAR S1a and the tetrapod TAAR1 subfamily was confirmed by their sequence similarities (70% to the mouse TAAR1), reciprocal blastp results, and phylogenetic analysis (Fig 2 and S1 Fig). The shark TAAR S2a was most similar to TAAR4 proteins (63% to the mouse TAAR4). However, this probably reflects the retention of ancestral characteristics by TAAR4 rather than orthology. Phylogenetic placement of TAAR S2a indicates that this shark TAAR gene diverged from the lineage leading to TAAR2-4 (Fig 2) or even all other gnathostome TAARs other than TAAR1 (S1 Fig).

The teleost fish genomes have generally higher numbers of the TAARs than the tetrapod genomes and the numbers vary significantly among teleost genomes (ranging from 18 in fugu to 110 in zebrafish) [29]. Our phylogenetic analysis showed that teleost TAARs are placed in three separate phylogenetic groups (Fig 2 and S1 Fig). While one group shows a clear ortholog relationship with the tetrapod TAAR1 subfamily, other two groups have unclear phylogenetic affinities. Hashiguchi and Nishida [26] also mentioned that the phylogenetic placement of these teleost clusters is not fully resolved. With multiple species-specific duplications and frequent loss of the TAAR-signature motif (S1 Fig, see also Materials and Methods), teleost lineages appear to have evolved TAAR subfamilies unique to their own and largely independent from tetrapod TAARs.

### Evolution of TAAR Subfamilies in Tetrapods

**Fig 3:**
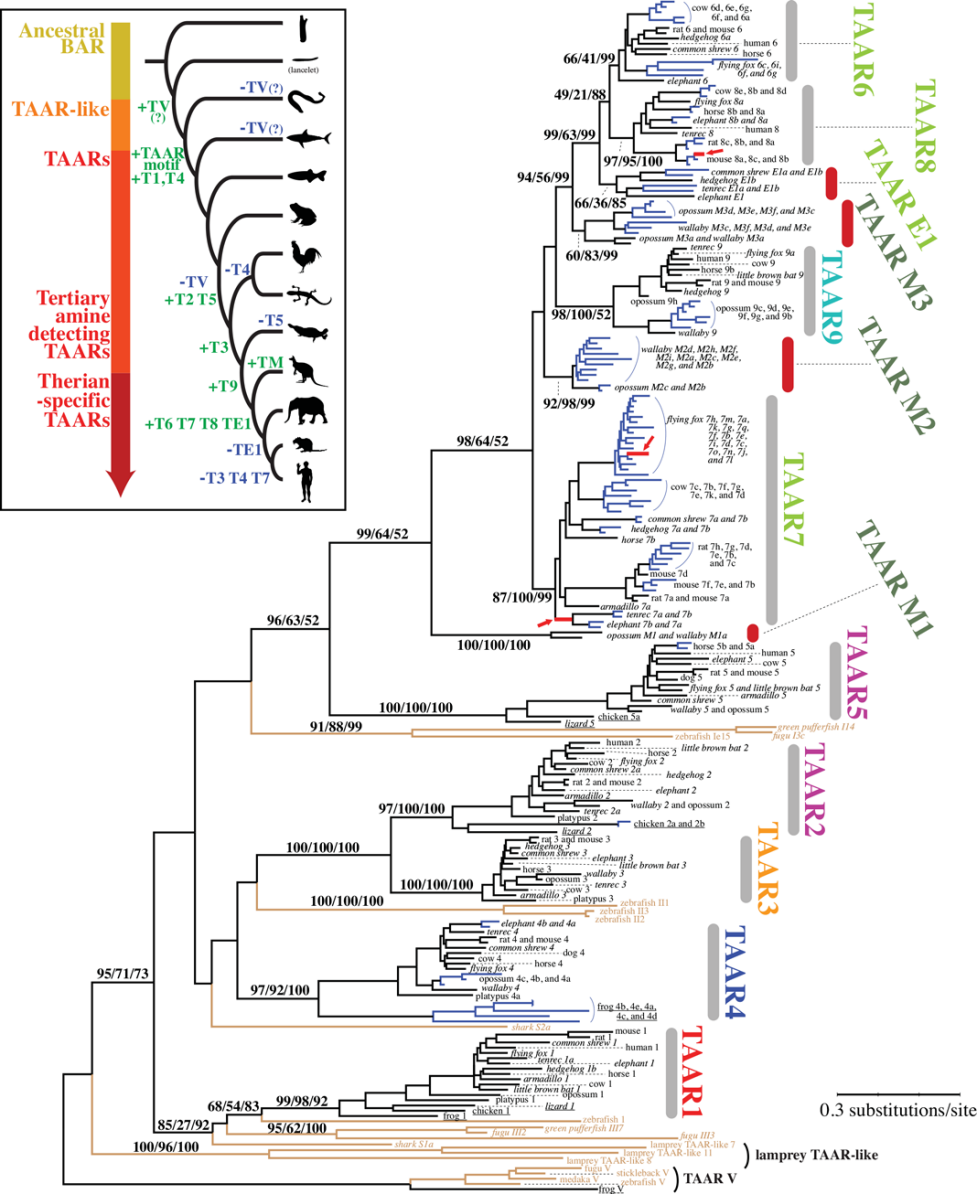
The maximum-likelihood phylogeny of TAAR proteins from 24 gnathostome genomes. All functional proteins in tetrapods, nine representative teleost proteins, and two elephant shark TAARs are included in the analysis. TAAR V as well as the lamprey TAAR-like sequences are used as the outgroup. The genes newly identified in this study are shown in italics. The numbers at internal branches show the bootstrap support values (%) for the maximum-likelihood and neighbor-joining phylogenies and the posterior probability (%) for the Bayesian phylogeny in this order. Supporting values are shown only for the major internal branches that have at least one method supporting higher than 70%. Blue-colored branches indicate the species-specific gene duplications within a cluster supported by higher than 80% of bootstrap values or posterior probability for all methods. Red-colored branches and arrows indicate those identified to be under positive selection by the branch-site models of PAML analysis (see S4 Table). Brown-colored branches indicate nine representative teleost TAARs, elephant shark TAARs, and lamprey TAAR-like proteins. The inset illustrates the evolution of vertebrate TAARs with approximate timing of various gain (green) and loss (blue) events. The vertebrate phylogeny is based on Blair and Hedges [33].

To gain further insights into the evolution of the TAAR subfamilies, we next restricted our attention to tetrapods with a focus on mammals, using the TAAR V genes from teleosts and frog as well as the TAAR-like sequences from the sea lamprey as the outgroups (Fig 3). All our phylogenetic analyses (Figs 2 and 3, and S1 Fig) support the TAAR1 subfamily representing the oldest divergence among the gnathostome TAAR lineages [26, 29]. This is consistent with its location at the beginning of the syntenic cluster (Fig 1) and its distribution across all vertebrates including fishes (Table 1). They have apparently remained as a single-copy gene in the majority of species analyzed. The remaining TAAR genes in the gnathostome subfamilies are grouped into two separate clades: one that includes the TAAR2-4 genes and the other that includes the TAAR5-9 genes as well as four newly defined mammalian-specific TAAR subfamilies (Fig 3). While there is no significant support for the phylogenetic placement of the shark TAAR S2a, as mentioned before, its position on the phylogenies would suggest that its ortholog gave rise to TAAR2-4 subfamilies (Figs 2 and 3) or probably all other TAARs (TAAR2-9, see S1 Fig). TAAR4 is probably the second oldest among the TAARs, or the oldest subfamily among the TAAR2-4 cluster, because TAAR4 sequences are found among mammals and frog (see Table 1 and Fig 1). It must have appeared prior to the split between amphibians and amniotes (reptiles, birds, and mammals) and had been subsequently lost in the common ancestor of reptiles and birds. The phyletic distribution and phylogenetic arrangement of the TAAR2 and TAAR5 genes would indicate that the origin of these subfamilies predates the origin of amniotes. Since TAAR2 and TAAR3 cluster together with a high bootstrap support (100%) and because of the presence of chicken and lizard TAAR2 genes, their origin must also predate the origin of amniotes. All other TAAR subfamilies in the phylogeny (TAAR6-9, M1-M3, and E1) form a monophyletic group and are restricted to mammals, suggesting that they are derived from a single-copy TAAR gene. In mammals, descendants from this gene duplicated multiple times to give rise to the TAAR6 to TAAR9 subfamilies as well as to four therian-specific subfamilies described in the next section (M1-M3 and E1).

In summary, we classify TAAR subfamilies into four separate groups based on the timing of their inferred emergence (see Fig 3 inset). TAAR1, the only TAAR that does not function as an olfactory receptor, is the oldest subfamily, as its origin probably predates the deepest split among gnathostomes. So far all TAARs except for TAAR1 have been found to be selectively expressed in olfactory epithelium. Thus the expression pattern changed after TAAR4 and newer TAARs diverged from TAAR1. TAAR4 is at least as old as tetrapods. Among other younger subfamilies, the origins of TAAR2 and TAAR5 are traced back to the common ancestor of amniotes, whereas all others are apparently derived from mammalian-specific duplications. Many of these timing estimates will have to be re-evaluated once detailed analyses of amphibian and sauropsid TAAR repertoires become possible.

In general, non-therian amniotes such as birds (*Gallus gallus* and *Taeniopygia guttata*), anole lizard (*Anolis carolinensis*), and platypus (*Ornithorhynchus anatinus*, Prototheria) have smaller numbers of TAAR genes than therian mammals (marsupials and placental mammals) (Table 1). Although based on the timing of their origins, these lineages would be expected to include members of five TAAR subfamilies, TAAR1-5, these genomes have retained only up to four subfamilies. Note also that the frog (*X*. *tropicalis*) genome has only copies of the two oldest types of TAARs (TAAR1 and TAAR4). The older types of TAAR subfamilies (TAAR1-5) exist as single-copy genes in each genome except for the expansion of TAAR4 in three genomes (frog, opossum, and elephant). In amniotes, in most instances for these older types of TAAR gene subfamilies, only one of the duplicated copies has remained functional, as in the case with tenrec TAAR1a/1bP and TAAR2a/2bP/2cP, hedgehog TAAR1a/1bP, and common shrew TAAR4a/4bP ('P' indicating a pseudogene). The two exceptions to this pattern are the chicken TAAR2a/2b and horse TAAR5a/5b where both duplicated genes have intact structures.

### Therian Mammal Specific TAAR Subfamilies

The more recently diverged TAAR subfamilies (TAAR6-9, M1-M3, and E1) are apparently restricted to therian mammals (eutherians and metatherians; Table 1, Figs 1 and 3, and S1 Fig). The cluster including these TAAR subfamilies is supported by 99% bootstrap value in the maximum likelihood phylogeny. These TAAR subfamilies must have emerged after the divergence between Prototheria (*e*.*g*., platypus) and Theria (230–166 MYA) [34, 35]. TAAR6-8 are all eutherian (placental mammal) specific. In addition, we found eutherian- and three metatherian (marsupial)-unique TAAR subfamilies (TAAR E1 and TAAR M1-M3, respectively) in this cluster. Three metatherian (tammar wallaby and opossum) TAAR groups are highly supported (>99% by at least one method; Fig 3). While TAAR M1 is a single-copy gene, TAAR M2 and M3 show species-specific expansions. Although the TAAR E1 subfamily is not highly supported (less than 70% bootstrap values in the maximum-likelihood and neighbor-joining phylogenies but 0.85 posterior probability in the Bayesian phylogeny), it forms a distinct cluster consistently in the three different phylogenetic reconstructions. TAAR E1 is found only in a few species of mammals: in two species of Laurasiatheria (common shrew and hedgehog) and in two species of Afrotheria (tenrec and african elephant) (see Table 1 for details). Therefore, TAAR E1 must have been present in early eutherians but have been lost in the ancestral lineage of Euarchontoglires (human, mouse, and rat) as well as in many Laurasiatheria species.

### Gain and Loss of TAAR Genes in Different Mammalian Lineages

The number of TAAR genes varies widely among the mammals we examined, ranging from 0 in dolphin to 26 in flying fox (Table 1). Frequent gene gains have occurred particularly in therian-specific TAAR genes (species-specific duplications are shown with blue branches in Fig 3).

As shown in Table 1 and Fig 1, the human genome does not have functional copies of TAAR3, TAAR4, and TAAR7. Stäubert *et al*. [36] showed that pseudogenization of TAAR3 and TAAR4 happened before the divergence of human and orangutan (for TAAR3) or gorilla (for TAAR4). Interestingly, they also showed that independent pseudogenizations have also occurred in the marmoset/tamarin lineages for both TAAR3 and TAAR4. Our preliminary search showed that in parallel to human, common marmoset (*Callithrix jacchus*) also lost TAAR7 (no pseudogene is found). In fact, the marmoset genome has only two functional TAAR genes: TAAR1 and TAAR5. All other five TAAR sequences we found were pseudogenes. Marmoset appears to have the fewest number of functional TAARs following dolphin and dog (Table 1). Fewer gene numbers in primates have been reported also for the OR gene family (S1 Table) [37, 38], which has been associated with poor olfaction senses in primate species [24].

The most extreme reduction in TAAR repertoire is seen in the bottlenosed dolphin (*Tursiops truncatus*) genome, which apparently has no functional TAAR gene, and only possesses three pseudogenes (TAAR1P, TAAR9aP, and TAAR9bP). As an interesting concordance, the dolphin appears to have also lost most but 26 of the functional OR genes (S1 Table) (also [24]). Our preliminary study shows that dolphin genome carries only three and four intact vomeronasal type-1 and type-2 receptor genes, respectively, and no functional gene but three pseudogenes of the Taste 1 (sweet taste) receptor. Massive losses of taste receptor genes have been also reported recently from toothed and baleen whales [39]. Dolphin, and cetaceans in general, appears to be a group of mammals that have the smallest number of chemoreceptors, apparently associated with their secondary adaptation for the aquatic environment and with the TAAR genes following the trend.

The dog genome has only two functional TAARs (TAAR4 and TAAR5) and two pseudogenes (TAAR1P and TAAR2P). On the contrary, a large number of OR genes (822 functional genes) with high divergence and with a small proportion of pseudogenes (25.3%) are found in the dog genome compared to other tetrapod species (S1 Table) [21, 40]. The TAAR1 pseudogenization seems to be a recent event. It must have happened after the divergence from feliforms because TAAR1s are all pseudogenes in wild gray wolf and four other caniforms but it is intact in cats [41]. The reliance on the higher number of ORs in the dog may have led to the reduction of TAARs due to their possibly overlapping functions.

The flying fox (*Pteropus vampyrus*) genome carries the largest number of TAARs (26 genes and 10 pseudogenes) while another Chiroptera, little brown bat (*Myotis lucifugus*), has a smaller number of TAARs (6 genes and 1 pseudogene). The larger number of TAARs in flying fox is caused, on one hand, by the flying fox-specific duplications of TAAR6 and TAAR7, and on the other hand, by the loss of TAAR6-8 in little brown bat. It is possible that the functions of TAAR6 and TAAR7 subfamilies may be related to dietary difference between fruit-eating flying fox and insectivorous little brown bat. TAAR7 especially is most prone to duplicate among TAAR subfamilies (Table 1 and Fig 3), and as described later, positive selection is detected in some TAAR7 genes. We should note, however, that no difference has been observed between these two Chiroptera species in terms of evolutionary patterns (*e*.*g*., selection and gene numbers) in other chemoreceptor genes such as sweet taste receptors [42], ORs [24], and vomeronasal sensitivity [43]. The sensory trade-off hypothesis has been considered for enhanced color-vision in primates and their often reduced or inactivated chemosensory genes ([44, 45], however [46]). A similar scenario may be considered for echolocating insectivorous little brown bat, which lost three TAAR genes. However, laryngeal echolocation appears to have evolved earlier than the divergence of the two Chiroptera species we examined [47], and as mentioned above, no such associated difference is known for other chemoreceptors in these or other Chiroptera species. It is thus difficult to apply the trade-off hypothesis in this case.

The numbers of OR and TAAR genes both vary widely among mammalian genomes (see S1 Table for the number of OR genes). In general, their numbers appear to be correlated. The dolphin genome has only 26 OR genes and no TAARs. Primates and platypus have relatively small numbers of OR as well as TAAR genes. Rodents (mouse and rat), cow, and opossum all have large numbers of both OR and TAAR genes. Exceptions are, as mentioned before, the dog genome where the majority of TAAR gene functions seem to have been displaced with highly divergent OR functions (more than 800 functional genes are found), and the two Chiroptera genomes where TAAR gene numbers vary significantly (6 *vs*. 26) while similar numbers of ORs are found between them. The two chemoreceptor families thus seem to have complex relationships in response to both environmental and evolutionary factors.

### Functional Differentiation among TAAR Subfamilies

TAARs are classified into two groups based on the types of ligands (amines) they detect [12]. TAAR1-4 are stimulated by primary amines (*e*.*g*., isoamylamine), which can be derived from natural amino acids by a single decarboxylation reaction. TAAR5-9, on the other hand, detect tertiary amines (*e*.*g*., *N*,*N*-dimethylated amines). We note that such TAAR ligand preference has been directly confirmed only in limited organisms (*e*.*g*., human and rodent). However, the amino acid sequences, especially those corresponding to ligand-binding sites, are highly conserved within each TAAR subfamily, and as our phylogenetic analysis clearly showed (Figs 2 and 3), proteins in TAAR5-9 cluster together and distinctively from those in TAAR1-4. Therefore, we hypothesize that TAAR proteins in each group share similar preference for tertiary *vs*. primary amine ligands. Following also Ferrero *et al*. [12], we call these two TAAR groups tertiary amine and primary amine detecting TAARs, respectively. The phylogenies indicated that the tertiary amine-detecting TAARs emerged from an ancestral type, primary amine-preferring TAAR.

The "differential tuning hypothesis" has been put forth to explain why tetrapods have two olfactory systems: the main olfactory system (MOS) and the vomeronasal system (VNS) [48, 49]. It is suggested that receptors expressed in MOS are broadly-tuned generalists that can detect an overlapping set of ligands and thus are more likely to be conserved, while receptors expressed in VNS are narrowly-tuned specialists and would evolve in a more lineage-specific manner. Grus and Zhang [48] tested this hypothesis and showed that VNS-expressed vomeronasal receptors (V1Rs and V2Rs) in tetrapods have abundant lineage-specific gene gains and losses. They found opposite patterns in MOS-expressed ORs and TAARs.

**Fig 4:**
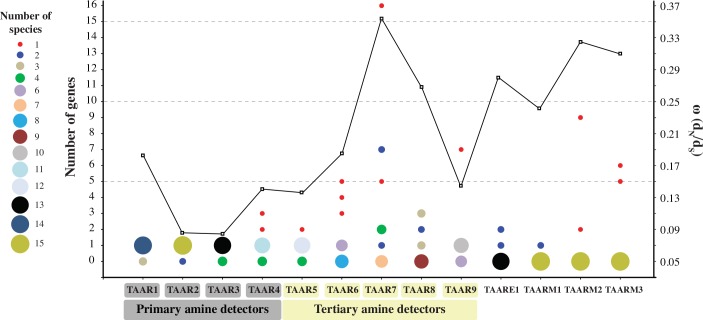
The number of TAAR genes within each TAAR subfamily for each therian species. The size of bubbles denotes the number of species where the corresponding TAAR genes are found. The average ω (dN/dS) calculated by the PAML M0 model for each TAAR subfamily is also plotted (open squares).

In our study, differences in evolutionary patterns were also found among the TAAR subfamilies. Fig 4 compares the number of TAAR genes among TAAR subfamilies for each therian species. While very few species-specific gene duplications were observed in primary amine-detecting TAAR subfamilies (TAAR1-4), multiple species-specific duplications were found in tertiary amine-detecting TAARs (TAAR5-9). Other newer TAAR subfamilies (TAAR E1 and M1-M3) belong to the same cluster with TAAR5-9. They are potentially tertiary amine detectors and also have multiple duplications. Grus and Zhang [48] observed two TAAR groups: TAAR1-5 and TAAR6-9 based on mouse, rat, and opossum data. With more data, our current analysis clarified the TAAR subfamilies to be classified into two groups that are consistent with their ligand types (primary *vs*. tertiary amines).

### Different Evolutionary Patterns in Primary and Tertiary Amine-Detecting TAARs

In order to test possible differences in evolutionary patterns between primary and tertiary amine-detecting TAARs, we estimated the average ω (the ratio of nonsynonymous to synonymous distances, d_N_/d_S_) for each TAAR subfamily. As shown in Fig 4 (see also S3 Table), the average ω's were about two times higher in tertiary amine detectors than in primary amine detectors (ω ranging from 0.0774 to 0.1807 for TAAR1-4 and from 0.1388 to 0.3512 for TAAR5-9, E1, and M1-M3; the difference between two groups is significant with *P* = 0.005 by one-tailed *t*-test and *P* = 0.0253 by Mann-Whitney *U* test). We selected four representative TAAR subfamilies: two primary amine detectors (TAAR1 and TAAR3) and two tertiary amine detectors (TAAR7 and TAAR8) and tested which lineage(s) show(s) significantly different ω using the PAML branch models [50]. Estimated ω's were significantly larger in TAAR7 compared to other lineages (*P* < 0.0001; Tests 1, 4, and 5 in S4 Fig). ω was also significantly larger in TAAR8 when compared against primary amine-detecting TAAR lineages (*P* = 0.0031; Test 2 in S4 Fig). Thus, the nonsynonymous substitutions in these tertiary amine-detecting TAAR subfamilies were substantially accelerated after the divergence from older primary amine-detecting TAARs. We next tested with the site models for the possibility of positive selection in each TAAR subfamily. The tests showed a highly significant support of positive selection for TAAR7 (*P* < 0.0001) and a weak but significant support (*P* = 0.0327) for TAAR8 (S4 Table). To further confirm the occurrence of positive selection in tertiary amine detectors, we tested using the branch-site models that can detect a short episode of positive selection occurring in a small fraction of amino acids [51]. Based on the results obtained above, we chose TAAR7 and TAAR8 for this test. As summarized in S4 Table, significant results were found in two branches in TAAR7 and one branch in TAAR8. These branches are also shown in red in Fig 3. It further supports that the evolution of tertiary amine-detecting TAARs has been partly driven by positive selection.

### Positive-Selection Sites Are Located in the Potential Ligand-Binding Sites in TAAR7 and TAAR8 Proteins

**Fig 5:**
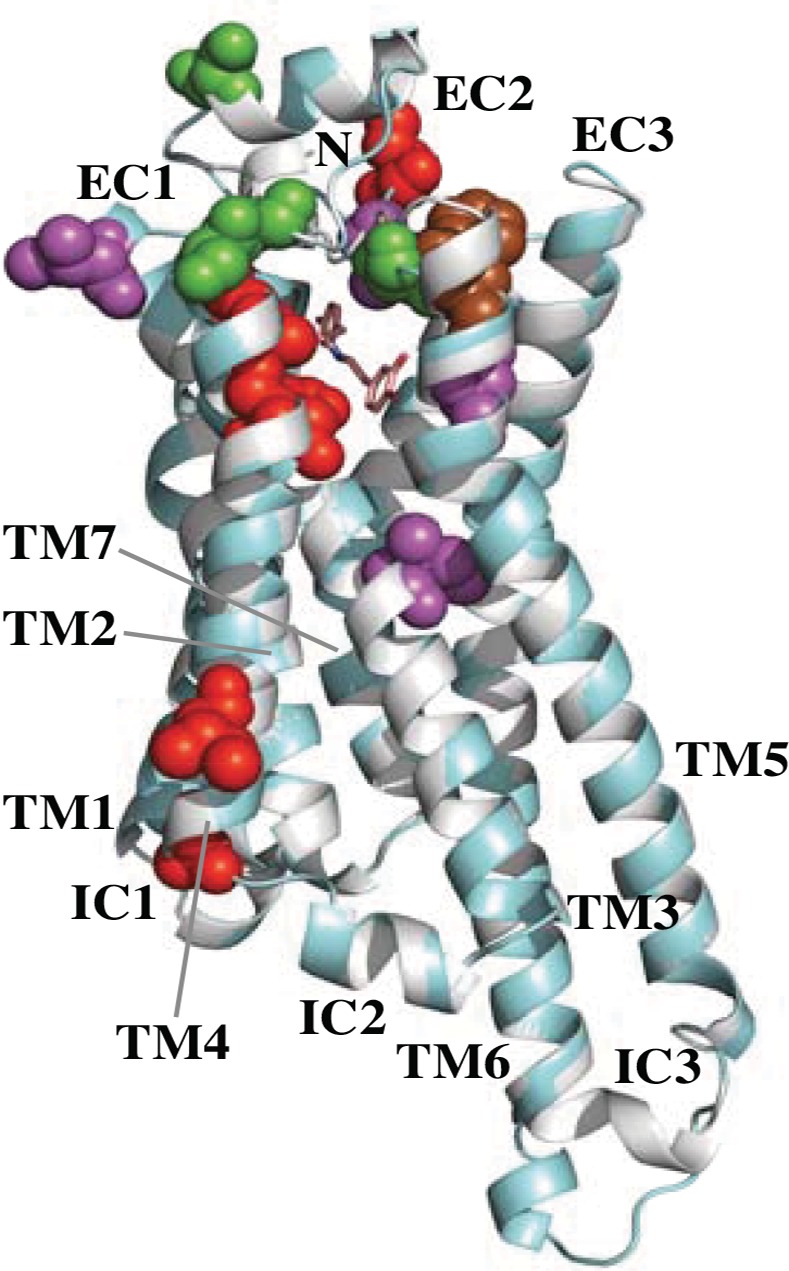
The 3D-structural model of the elephant TAAR7a protein (cyan) superimposed with the turkey β1-adrenergic receptor (β1AR, gray). The ligand of the β1AR, dobutamine, is shown with the stick model. Positively selected sites are indicated by red (detected by the site model in TAAR7), green (detected by the branch-site model in flying fox TAAR7c and elephant TAAR7a), purple (detected by the site model in TAAR8), and brown (detected by the branch-site model in mouse TAAR8a). The transmembranes (TM) and internal/external loop (IC1-3 and EC1-3) regions as well as N-terminal (N) are labeled. The C-terminal is invisible locating behind TM1. See S6 Fig for more details.

For TAAR7 and TAAR8, the amino acid sites under positive selection were identified using the Bayes Empirical Bayes (BEB) inference [52]. Eleven sites were identified with the site models (S3 Table) and six sites with the branch-site models (S4 Table). Four of eleven sites identified in TAAR7 (positions 137^4.39^, 155^4.57^, 184, and 188^5.36^; see Materials and Methods and S5 Fig for the Ballesteros and Weinstein numbering scheme shown as superscripts) and one of five sites identified in TAAR8 (position 194^5.42^) had their posterior probabilities higher than 0.95, a strong indication of positive selection. The spatial distribution of these sixteen positive-selection sites on the TAAR proteins is illustrated in Fig 5 (see S6 Fig for more details). Thirteen sites are present in the extracellular loop regions, especially in EC2, and in the extracellular-ends of TM regions. As shown in S5 Fig, many residues predicted to involve with ligand-binding based on the solved protein structures are distributed in EC2, EC3, and their surrounding areas. The positive-selection sites are concentrated especially in the area surrounding the predicted main ligand-binding pocket. The seven positively selected sites in TAAR7 and TAAR8 (positions 103^3.32^, 104^3.33^, 159^4.61^, 184, 186, 190^5.38^, and 194^5.42^) correspond to residues identified to be directly involved with ligand-binding on related biogenic amine receptors, β-adrenergic receptors 1 and 2 (β_1_AR and β_2_AR) [53–55] (see S5 Fig for the details). Positions 104^3.33^ and 155^4.57^ were identified to be under positive selection in TAAR7 (S3 Table, and S5 and S6(C) Figs).

A mutational study of the β_2_AR demonstrated that replacement of two amino acids (corresponding to positions 151^4.53^ and 155^4.57^ in human TAAR1) significantly affected the receptor expression and agonist-stimulated activity [56]. The position 104^3.33^ is usually conserved with valine in βARs. However, a mutation in 104^3.33^, which by itself reduces the ligand-binding affinity, was found to rescue the binding affinity in double mutants. Different residues in this position were reported to affect the binding of both agonists and antagonists [57]. Therefore, these positive-selection positions are potentially important in functions including the folding and ligand-binding.

Ferrero *et al*. [12] demonstrated that mutating two amino acids closely located to possible ligand-binding sites in TM3 (108^3.37^ and 109^3.38^) between those found in the mouse TAAR7e (SS) and those in TAAR7f (YC) dramatically reversed the ligand responsiveness. In our PAML site-model (M8) analysis of TAAR7, these two sites have relatively high ω's (1.022 and 0.902) but low posterior probabilities (< 0.3). Other sites whose ω's were larger than 1.0 but have low posterior probabilities include 100^3.29^ and 196^5.43^. The position 100^3.29^ is one of the ligand-binding sites (S5 Fig). Two other ligand-binding neighboring sites, 28^1.37^ and 152^4.54^, in TAAR8 were also identified with high ω's (site-model M8 analysis) but with low probabilities. Although they may be false positives, it warrants further studies.

### Changes of Amino Acid Properties in Positive-Selection Sites

Many amino acid changes found in the positively selected sites are those altering physicochemical properties (S7 Fig). We examined these substitutions using TreeSAAP [58, 59]. Side-chain changes involving volume, torsion angles, hydrophobicity, and charge found in positively selected positions as well as their neighboring sites were shown to be under positive destabilizing selection (*P* < 0.001). Pairwise TreeSAAP analysis also showed that many long branches found in the TAAR7 family (*e*.*g*., flying fox 7h and cow 7c in Fig 3) may also be under such positive destabilizing selection. Of particular interests is three changes identified in the tenrec/elephant lineage of TAAR7 using branch-side models. All three changes (positions 161, 177, and 188^5.36^) involve acquisition of serine residues. Changes involving serines are also found in two other highly significantly supported positions (155^4.57^ in TAAR7 and 194^5.42^ in TAAR8). All these changes are located within or at the border of the EC2 loop region. Although the positions are not consistent, for β_1_AR, serine residues in TM5 (positions 194^5.42^, 195^5.43^, and 198^5.46^) have been reported to be critical for agonist binding and receptor activation [60, 61]. Structural analysis of β_1_AR by Warne *et al*. [53] indicated that the ligand-induced rotamer conformational changes of these serine residues and stabilization of the contracted ligand-binding pocket (through hydrogen-bonding interactions between the ligand and these residues) dictate the efficacy of ligands. Therefore, the changes found in these positive-selection sites may have played an important role in defining ligand-binding activities and specificities among proteins belonging to the tertiary amine-detecting TAAR subfamilies.

### Ligand-Binding Sites Show Different Evolutionary Patterns

The ligand-binding space in the Rhodopsin-like GPCR proteins consists of a deeper main ligand-binding crevice and a shallower minor binding pocket [62, 63]. The latter area is considered to be important for receptor activation rather than ligand-specificity. The residues surrounding the minor pocket are in fact highly conserved especially among TAARs (S5 Fig) and consistent with potentially higher selective constraints. Interestingly, the position 103^3.32^ in TM3 was found to be under positive selection in TAAR7, and it is located at the boundary between the two binding pockets. Kleinau *et al*. [55] showed that six of the twenty nine residues identified as ligand-binding sites are conserved among biogenic amine receptors including human TAARs and adrenergic receptors, and considered them to be determinants of the ligand-binding regions among these receptors. All but one (103^3.32^) of these positions are in fact highly conserved among the TAARs we examined. Kleinau *et al*. [55] further pointed out that six additional ligand-binding residues in human TAAR1 are identical or similar to those of biogenic amine receptors. They speculated that this similarity could explain the ligand promiscuity of TAAR1. While we confirmed that these residues are also conserved in all other TAAR1s, residues in the corresponding positions in tertiary amine-detecting TAARs are more diverse (see S5 Fig for the details).

## Conclusion

Our molecular evolutionary analysis of metazoan TAARs showed that an ancestral-type TAAR-like protein emerged in lamprey. The conserved TAAR signature motif appeared after jawed vertebrates diverged from jawless fish. Among mammalian TAARs, older types of TAAR subfamilies (TAAR1-4) are primary amine-detecting receptors. They are more conserved and maintained as single-copy genes in each genome except for TAAR4. Newer types of mammalian TAARs (TAAR5-9, M1-M3, and E1) are considered to be tertiary amine-preferring receptors. They are found only in therian mammals and, except for TAAR5, have experienced frequent species-specific duplications. Our evolutionary analysis found evidence of positive selection distributed around the ligand-binding sites in TAAR7 and TAAR8 proteins. These changes could have affected ligand-binding activities and specificities in these TAARs. It may have contributed to therian mammal's adaptation to the dynamic land environments by allowing finer discrimination among a diverse array of volatile amines. Specific ecological conditions in some species may have led to additional duplications or losses of especially tertiary amine-detecting TAARs. Furthermore, birth and death processes of two chemoreceptor families (ORs and TAARs) seem to be under the influence of both environmental and evolutionary factors. Further studies on TAAR evolution and their functions will provide more insights into functional divergence of chemosensory receptors.

## Materials and Methods

### Query and Genome Sequences

Previously reported TAAR genes were used as search queries. The sequences were obtained from Lindemann *et al*. [15] and from Hashiguchi and Nishida [26]. Genomic sequences were obtained from multiple sources (S1 Table). It includes 17 mammals (14 eutherians, 2 metatherians, and 1 prototherian), two birds, one reptile, one frog, two teleost fishes, elephant shark, as well as four non-vertebrate species. Note that the zebrafish TAARs and sea lamprey TAAR-like genes obtained from Hashiguchi and Nishida [26] are also included in our analysis.

### TAAR Gene Mining

Similarity search was performed using the Basic Local Alignment Search Tool (BLAST, ver. 2.2.17) programs [64]. The default parameters were used for tblastn except for setting the effective length of database (option -z) to 1.1×10^10^. This was done to obtain E-values comparable among different sizes of genomes and equivalent to those from the search against the non-redundant (NR) protein database at the National Center for Biotechnology Information (NCBI, http://www.ncbi.nlm.nih.gov). The E-value threshold of 1×10^−30^ was used to identify TAAR gene candidates from each genome. The putative TAAR genes were verified by searches using blastp against the NR database. A putative protein was considered to be a TAAR candidate if the top hit from the blastp search was a previously known TAAR. The TAAR candidates newly identified were subsequently used as queries against their genomes again to find any additional candidates. These steps were recursively performed until no other TAAR candidate sequences were detected from each genome.

One group of TAAR genes, designated as the TAAR subfamily V (or simply "TAAR V") by Hashiguchi and Nishida [26], has been identified only from a limited number of species, mostly from teleost fishes. For more sensitive search, we built a profile hidden Markov model (HMM) with five TAAR V protein sequences from frog (*X*. *tropicalis*, XP_002935532), zebrafish (*Danio rerio*, XP_001337671), spotted green pufferfish (*T*. *nigroviridis*, CAF93600), stickleback (*Gasterosteus aculeatus*, [26]), and medaka (*Oryzias latipes*, [26]). Each genome was searched using the hmmbuild and hmmsearch programs of the HMMER package (ver. 3.0) [65] with default parameters.

The TAAR genes are intron-less and encoded in a single exon. TAAR2 genes, also known as GPR58, are exceptions and have two exons. To determine exon-intron boundaries for TAAR2, a profile HMM was built from human, mouse, and rat TAAR2 protein sequences using the HMMER package (ver. 2.3.2) [66]. Using this profile HMM, the coding sequences were predicted using GeneWise (ver. 2.2) [67].

### TAAR Signature Motif

TAAR proteins have a unique peptide motif that is absent from all other known GPCRs [15]. This motif is located within the seventh transmembrane (TM) region, and defined as NSX_2_NPX_2_[Y/H]X_3_YXWF where X_n_ represents any n amino acid residue(s) (S2(A) Fig). The motif is most strongly conserved in the TAAR3 family (S2(B) Fig). All tetrapod TAAR proteins identified in this study have this motif, while all lamprey TAAR-like and five TAAR V proteins have only weakly conserved motifs. Motifs found in the corresponding regions of the lamprey TAAR-like and TAAR V proteins are XSX_2_NPX_2_[Y/F]X_6_F and NSX_2_NPX_2_YX_3_[H/N]XS[Y/F], respectively. Among the 157 teleost TAARs we identified, 32 of them from zebrafish and green pufferfish have only weakly conserved TAAR signature motif (S2(C) Fig). We also found 9 teleost TAARs that lack the signature motif. In S1 Fig, the distribution of teleost fish TAARs among vertebrate TAARs as well as the conservation of the motif is illustrated.

### Multiple Sequence Alignments

Multiple alignments of TAAR protein sequences were generated using MAFFT with the L-INS-i algorithm (ver. 6.24) [68], MUSCLE (ver. 3.7) [69], ProbCons (ver. 1.12) [70], and PRALINE [71], each with the default parameters. Alignments were adjusted manually when necessary. For consistency, all amino acid positions shown in this study are numbered based on the human TAAR1 sequence in the alignment given in S5 Fig. Position numbers are also presented using the scheme proposed by Ballesteros and Weinstein [72]. In the Ballesteros-Weinstein system, the most conserved residue in each TM region among all Rhodopsin-class GPCRs is assigned the position index “50” and the rest of the positions within each TM region are numbered accordingly. In this study the Ballesteros-Weinstein position numbers are based on the TM regions of the turkey β_1_-adrenergic receptor (β_1_AR, P07700) sequence obtained from the GPCRDB Web server (http://www.gpcr.org/7tm) [73]. These numbers are given as superscripts. All TAAR sequences and alignments are available in: http://bioinfolab.unl.edu/emlab/TAAR

### Phylogenetic Analysis

Phylogenetic relationships were reconstructed by the maximum-likelihood method with the PROTGAMMAJTT model (JTT matrix with gamma-distributed rate variation) using RAxML (ver. 7.0.4) [74]. The neighbor-joining phylogenies [75] were reconstructed by using neighbor of the Phylip package (ver. 3.67) [76]. The protein distances were estimated using protdist of the Phylip package with the JTT substitution model with the gamma-distributed rate variation (α = 1.3004 was estimated using the maximum-likelihood method implemented RAxML) [77]. Bayesian inference of phylogeny was performed using MrBayes (v3.1.2) [78] with the JTT substitution model with the gamma-distributed rate variation (α = 1.3004). The Markov chain Monte Carlo search was run for 10^6^ generations, with a sampling frequency of 10^3^, using three heated and one cold chain and with a burn-in of 10^2^ trees. In addition to TAAR sequences, eight representative biogenic amine receptors (BARs), four cow opsin sequences, as well as eight representative dog ORs were included in phylogenetic analysis. OR sequences were used as the outgroup. Non-parametric bootstrapping with 1000 pseudo-replicates [79] was used to estimate the confidence of branching patterns for the maximum-likelihood and neighbor-joining phylogenies. Presentation of the phylogenies was done with FigTree (http://tree.bio.ed.ac.uk/software/figtree). All phylogenies are available in: http://bioinfolab.unl.edu/emlab/TAAR.

### Transmembrane Protein Topology Prediction

HMMTOP (ver. 2.1) [80] and Phobius (ver. 1.01) [81] were used to predict the transmembrane protein topology, which includes N-terminal, transmembrane (TM), intercellular loop (IC), extracellular loop (EC), and C-terminal regions.

### Tests of Selection Patterns

Selection patterns were tested using the maximum-likelihood framework developed by Goldman and Yang [82]. The site-, branch-, and branch-site models implemented in codeml of the PAML (Phylogenetic Analysis by Maximum Likelihood) package (version 4.5) were used [50]. We first used the site-model M0 (one-ratio, ω, for all sites) to estimate the d_N_/d_S_ (ω) for each TAAR subfamily. Two sets of likelihood-ratio tests (LRTs; d.f. = 2) were performed for positive selection: M1a (two site-classes, nearly neutral model: 0 < ω_0_ < 1 and ω_1_ = 1) *vs*. M2a (three site-classes including positive selection: 0 < ω_0_ < 1, ω_1_ = 1, and ω_2_ > 1) and M7 (beta distribution and 0 < ω < 1) *vs*. M8 (beta distribution and ω > 1). Using the branch models, we performed LRTs with d.f. = 1 between a one-ratio model (R1; the same ω for all branches) and a two-ratio model (R2; two independent ω's) [83, 84]. As illustrated in S4 Fig, each test was set to compare primary amine-detecting TAAR lineages (TAAR1 and TAAR3) against tertiary amine-detecting receptor lineages (TAAR7 and TAAR8). We also used the branch-site models in order to detect positively selected sites along specific branches [51, 84]. In these models, positive selection was allowed on a specific, "foreground", branch, and the LRTs (d.f. = 1) were performed against null models that assume no positive selection. The branch-site test of positive selection ("Test 2" in [51]) has four site classes: 0, 1, 2a, and 2b. For the site classes 0 and 1, all codons are under purifying selection (0 < ω_0_ < 1) and under neutral evolution (ω_1_ = 1), respectively, on all branches. For the site classes 2a and 2b, positive selection is allowed on the foreground branches (ω_2_ ≥ 1) but the other, "background", branches are under purifying selection (0 < ω_0_ < 1) and under neutral evolution (ω_1_ = 1), respectively. For the null model, ω_2_ is fixed as 1. For our analysis, TAAR7 and TAAR8 subfamilies were tested. For each subfamily phylogeny, tests were done using each branch (from both internal and terminal branches) as the foreground. The numbers of tests performed were 61 and 26 for TAAR7 and TAAR8, respectively.

All PAML analyses were carried out using the F3X4 model of codon frequency [82]. The level of significance (*P*) for the LRTs was estimated using a χ^2^ distribution with given degrees of freedom (d.f.) and the test statistic calculated as twice the difference of log-likelihood between the models (2∆*ln*L = 2[*ln*L_1_ –*ln*L_0_] where L_1_ and L_0_ are the likelihoods of the alternative and null models, respectively). Positively selected amino acid sites are identified based on Bayes Empirical Bayes posterior probabilities [52].

### Analysis of Selection on Amino Acid Properties

Possible selection on changes in amino acid properties were examined by TreeSAAP (version 3.2) [58, 59]. The program reconstructs the ancestral character states at each node based on a given phylogeny. Observed amino acid substitutions are analyzed in the context of 539 physicochemical properties (downloaded from http://dna.cs.byu.edu/treesaap) [85] and their magnitude of change (in 8 categories, with 1 being the most conservative and 8 the most radical). Based on the methods by Xia and Li [86], McClellan and McCracken [87], and McClellan *et al*. [59], observed differences are compared against the expected differences under the neutrality. The most radical changes (categories 6–8) with significant positive z-scores (> 3.09; *P* < 0.001) are considered to be under positive-destabilizing selection. In order to confirm if the results are not affected by the phylogenetic topologies we used, we also performed pairwise analysis of TreeSAAP. Pairwise comparisons were done for 16 flying fox TAAR7, 29 other mammalian TAAR7, and 16 TAAR8 sequences. TreeSAAP results are available in: http://bioinfolab.unl.edu/emlab/TAAR.

### Protein Structural Homology Modeling

Homology modeling of TAAR protein structures was performed using the SWISS-MODEL Web server (http://swissmodel.expasy.org) [88]. The same template, the B-chain of the turkey (*Meleagris gallopavo*) β_1_AR (4AMJ), was selected for the human TAAR1, elephant TAAR7a, and mouse TAAR8a proteins. See S6 Fig for the details on TAAR protein structural modeling. The graphical representation of TAAR structures was prepared with the PyMOL Molecular Graphics System (version 1.3, Schrödinger, LLC).

## Supporting Information

S1 Fig The maximum-likelihood phylogeny of TAAR proteins from 25 vertebrates. (PDF)

S2 Fig TAAR signature motifs from TAAR subfamilies (a), from the TAAR3 subfamily (b), and from weakly conserved fish TAARs (c).
(PDF)

S3 Fig TAAR signature motifs found in the two elephant shark (*Callorhinchus milii*) TAAR protein sequences. (PDF)

S4 Fig PAML branch-model tests between primary amine-detecting TAARs (TAAR1 and TAAR3) and tertiary amine-detecting TAARs (TAAR7 and TAAR8). (PDF)

S5 Fig Multiple alignment of the four TAAR and the turkey β_1_-adrenergic receptor proteins. (PDF)

S6 Fig Modeling of the 3D-structure of TAAR proteins. (PDF)

S7 Fig Alignments of the positively selected sites identified in TAAR7 (a) and TAAR8 (b). (PDF)

S1 Table The animal genomes used in this study. (PDF)

S2 Table TAAR sequences used and identified in this study. (XLSX)

S3 Table The results of PAML site-model analysis for TAAR subfamilies. (PDF)

S4 Table The results of PAML branch-site model analysis. (PDF)
