# Supplementary material for: Molecular Evolution and Functional Divergence of Trace Amine–Associated Receptors
Source: PLoS One. 2016 Mar 10;11(3):e0151023. doi: 10.1371/journal.pone.0151023 (PMC4786312; doi:10.1371/journal.pone.0151023)
Supplement: S1 Fig — (PDF) [file pone.0151023.s001.pdf]

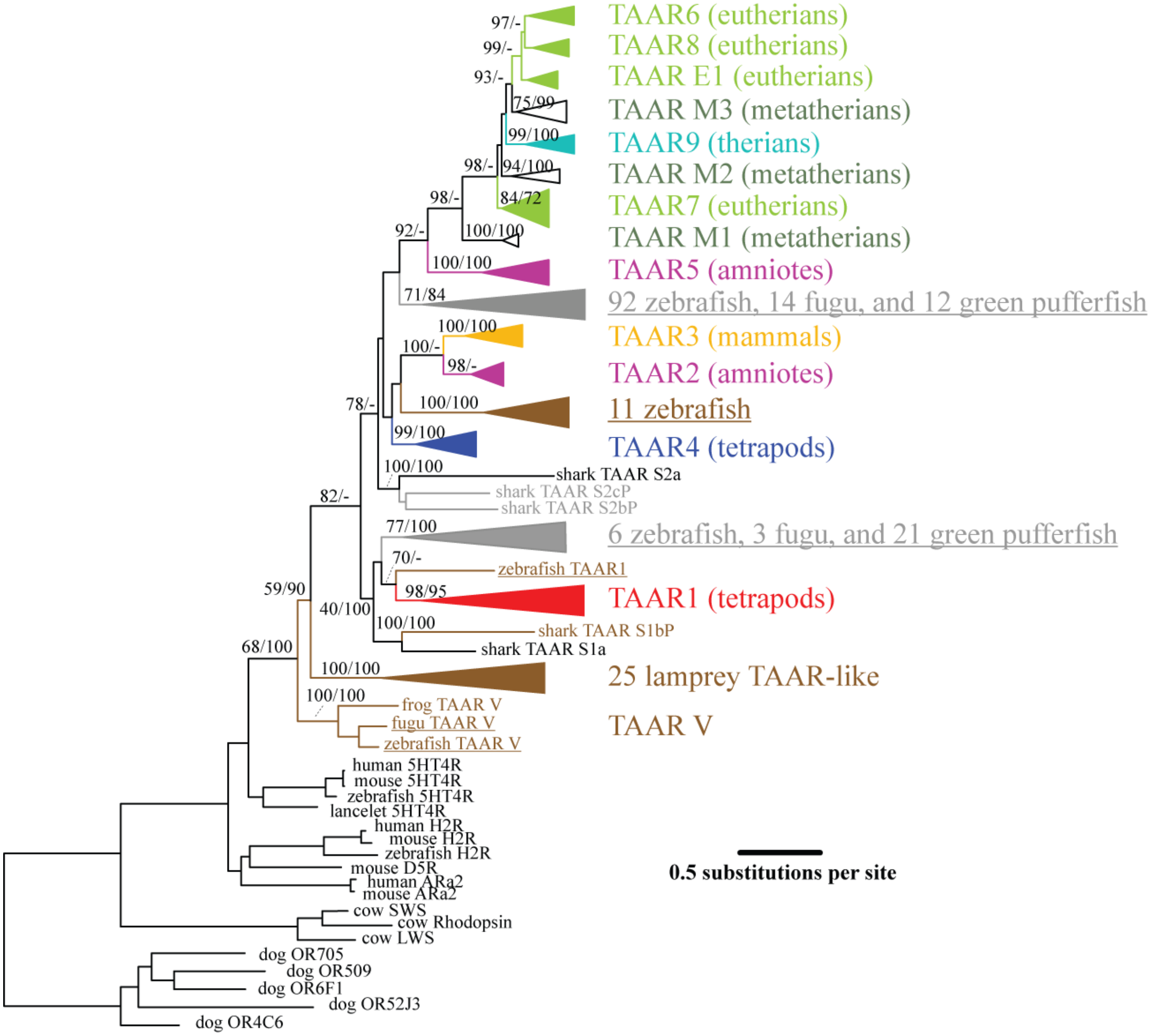

S1 Fig. The maximum-likelihood phylogeny of TAAR proteins from 25 vertebrates. Ten representative biogenic amine receptors (5HT4R: serotonin receptors, H2R: histamine receptors, D5R: dopamine receptors, and ARa2: adrenergic receptors), three cow opsins, and five representative dog olfactory receptors (ORs) are included as the outgroup. The numbers at internal branches show the bootstrap support values (%) for the maximum-likelihood phylogeny and the posterior probability (%) for the Bayesian inference phylogeny. Support values are shown only for the major internal nodes. Three metatherian-specific and one eutherian-specific TAAR groups are indicated as TAAR M1-M3 and TAAR E1, respectively. Teleost fish proteins are indicated with underline. Brown-colored branches indicate the protein lineages where all proteins have weakly conserved TAAR signature motifs (S2 Fig, see Materials and Methods). Two teleost fish clusters colored in gray have TAARs with mixed types of motifs: conserved, weakly conserved, or lost. Note also that the phylogenetic placement of these teleost fish clusters is not resolved. The expanded phylogeny showing all gene names is available on the website: http://bioinfolab.unl.edu/emlab/TAAR/
