# Supplementary material for: Molecular Evolution and Functional Divergence of Trace Amine–Associated Receptors
Source: PLoS One. 2016 Mar 10;11(3):e0151023. doi: 10.1371/journal.pone.0151023 (PMC4786312; doi:10.1371/journal.pone.0151023)
Supplement: S2 Fig — TAAR signature motifs from TAAR subfamilies (a), from the TAAR3 subfamily (b), and from weakly conserved fish TAARs (c). (PDF) [file pone.0151023.s002.pdf]

(a)
(b)
(c)
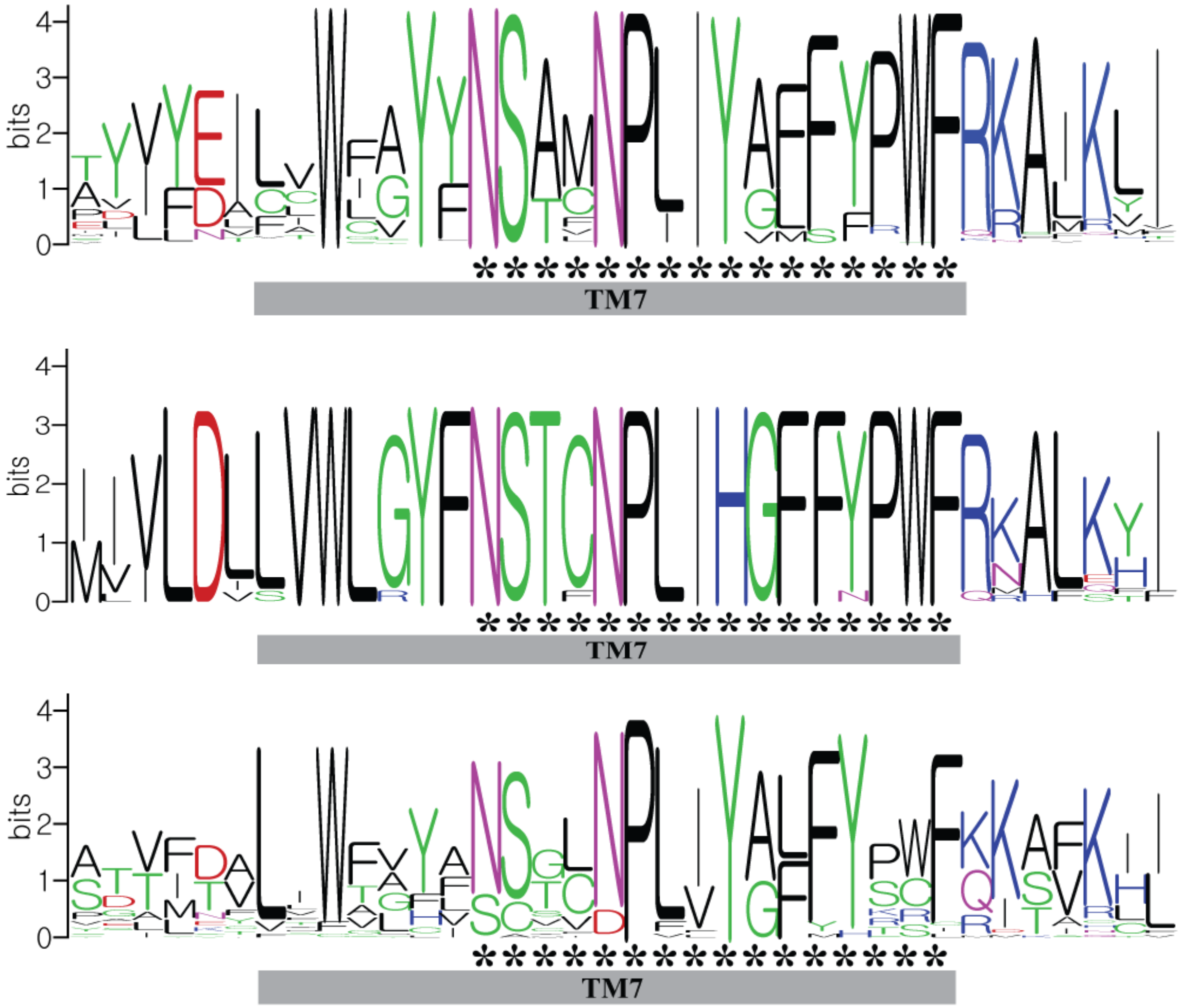

S2 Fig. TAAR signature motifs from TAAR subfamilies (a), from the TAAR3 subfamily (b), and from weakly conserved fish TAARs (c). Conserved amino acid patterns (Lindemann *et al.*, 2005) based on the multiple sequence alignments from positions 291 – 326 (numbering according to the mouse TAAR3: NP_001008429) are shown using the sequence logo (http://weblogo.berkeley.edu) (Crooks *et al.*, 2004). 209 sequences from TAAR1-9, M1-M3, and E1 (a) and 13 sequences from TAAR3 (b) were included in each multiple alignment. For fish TAARs, the sequence logo was generated using only 32 sequences where TAAR signature motif (NSX$_2$NPX$_2$[Y/H]X$_3$YXWF) is not conserved. The height of each amino-acid letter is proportional to its frequency of occurrence in a given position. The known TAAR signature motif (NSX$_2$NPX$_2$[Y/H]X$_3$YXWF) corresponds to the positions marked with *. The location of the seventh transmembrane region (indicated as TM7) was predicted using Phobius (Kall *et al.*, 2007).

References:

Crooks GE, Hon G, Chandonia JM, Brenner SE. 2004. WebLogo: a sequence logo generator. *Genome Res*. 14:1188-1190.

Lindemann L, Ebeling M, Kratochwil NA, Bunzow JR, Grandy DK, et al. (2005) Trace amine-associated receptors form structurally and functionally distinct subfamilies of novel G protein-coupled receptors. *Genomics* 85: 372-385.

Kall L, Krogh A, Sonnhammer ELL. 2007. Advantages of combined transmembrane topology and signal peptide prediction-the Phobius web server. *Nucleic Acids Res*. 35:W429-432.
