# Supplementary material for: Molecular Evolution and Functional Divergence of Trace Amine–Associated Receptors
Source: PLoS One. 2016 Mar 10;11(3):e0151023. doi: 10.1371/journal.pone.0151023 (PMC4786312; doi:10.1371/journal.pone.0151023)
Supplement: S3 Fig — (PDF) [file pone.0151023.s003.pdf]

>shark TAAR S1a

LCYESVNGSCPRAIRSTGVRITLYLLAVLAILVTLFGNMLVIISIAHFKQLHTPTNYLVF
SLAIADFLLGCIVMPYSLIRSIESCWYFGILFCKLHTSFDLVLCAASIIHLCCISVDRYY
AVCDPLKYKTTITVSTVLIMICLSWALSFLVGFVIIFLELHLIEIKDFYYHEIACFGGCT
LMMGKVCALVYSTISFYFPAFIMVCIYTKIYLVAKKQARTINNLSRKVQPINEGNSIASQ
RSERKAAKTLGIVMGVFILCWSPYFVCDSIEPFIKYSTPPVLFDAFFWVGYL**NSTFNPMI**
YGFFYSWFRKALKIILTCKIFAPDSSRINLF

>shark TAAR S2a

MNSINLENSEDLQYCFEFNMSCPKSIRSTTTTVTMYIFITISIVITILGNSVVMISILHF
KQLQTPTNYLVLSLAFVDFLMGFFVLPFSMVRSVETCWYFGDTFCDIHSTLDVVLTTVSI
YNLCFIAIDRYYAVCEPLLYSIKMTLPMTALIITLNWLFAIIYGSCVFLSEFTKKASGHY
RTTISCKGSCIEYRFGGHMDALIVLFIPTFIILGIYLKIYFVQRKHARKIGNMPNNINSK
EEINVRVLQTKEKTAAKNQGVVMGIFVLSWLPFYLSSIINPYLNFATPPILFEAFTWFGF
F**NSAFNPVLYAFFYPWF**RTALKSILTCQILRPESSIMNLFPE

S3 Fig. TAAR signature motifs found in the two elephant shark (*Callorhinchus milii*) TAAR protein sequences. The TAAR motif regions are highlighted with yellow. The seven transmembrane regions predicted by Phobius (Kall *et al.*, 2007) are indicated with underline.

Reference:

Kall L, Krogh A, Sonnhammer ELL. 2007. Advantages of combined transmembrane topology and signal peptide prediction-the Phobius web server. *Nucleic Acids Res*. 35:W429-432.
