# Supplementary material for: Molecular Evolution and Functional Divergence of Trace Amine–Associated Receptors
Source: PLoS One. 2016 Mar 10;11(3):e0151023. doi: 10.1371/journal.pone.0151023 (PMC4786312; doi:10.1371/journal.pone.0151023)
Supplement: S4 Fig — (PDF) [file pone.0151023.s004.pdf]

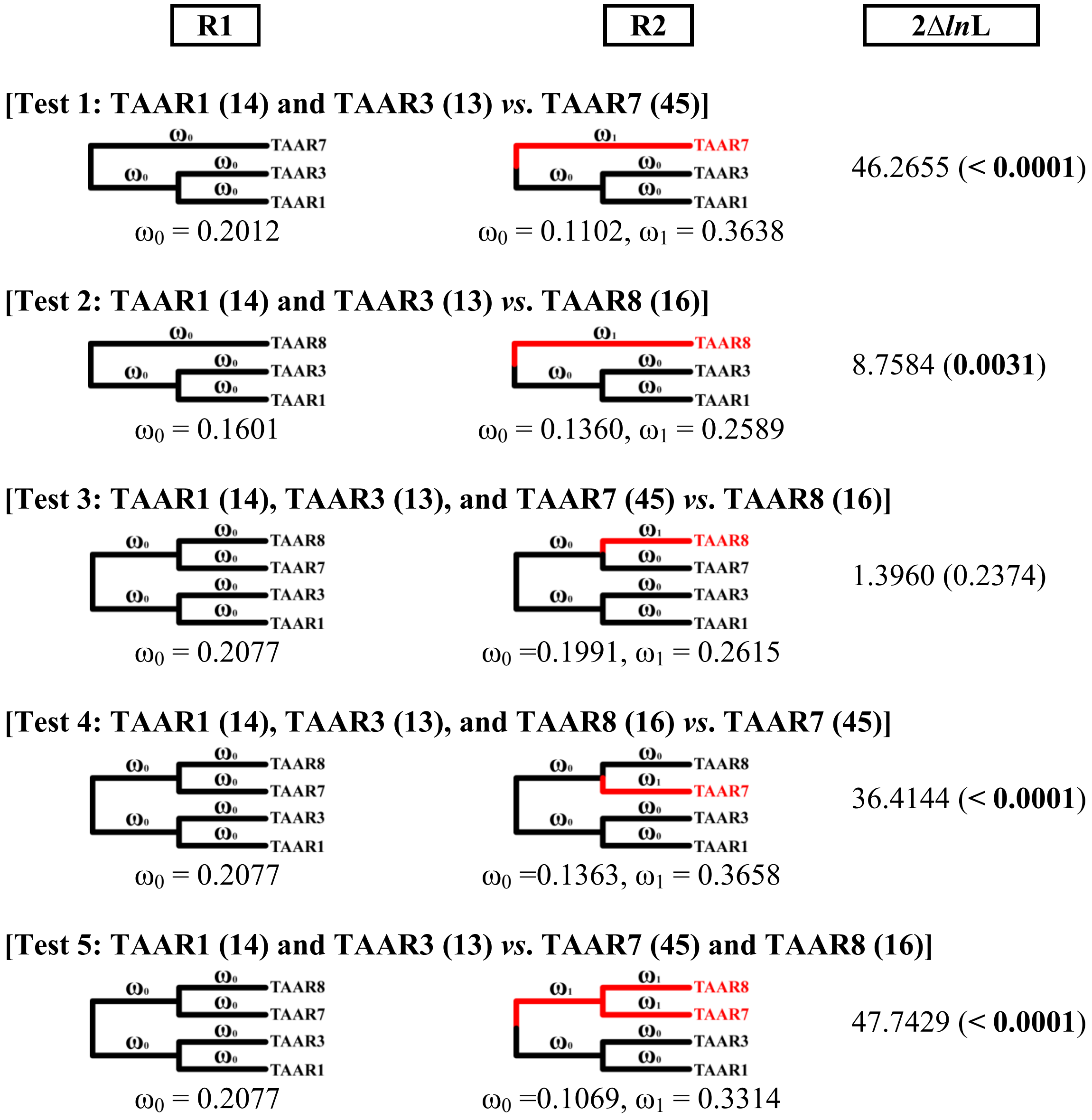

S4 Fig. PAML branch-model tests between primary amine detecting TAARs (TAAR1 and TAAR3) and tertiary amine detecting TAARs (TAAR7 and TAAR8). All tests were performed comparing the two hypotheses: R1 (a single ω for all branches) and R2 (two independent ω's: ω_1 for the red lineage and ω_0 for the black lineages). The number of the genes included in each TAAR subfamily is given in parentheses after the subfamily name. For the likelihood ratio test statistics, $2\Delta ln\mathrm{L}$, *P*-values (shown in parentheses) are obtained based on a χ^2 distribution with d.f. = 1. Significant *P*-values (< 0.05) are shown in boldfaces.
