# Supplementary material for: Molecular Evolution and Functional Divergence of Trace Amine–Associated Receptors
Source: PLoS One. 2016 Mar 10;11(3):e0151023. doi: 10.1371/journal.pone.0151023 (PMC4786312; doi:10.1371/journal.pone.0151023)
Supplement: S5 Fig — (PDF) [file pone.0151023.s005.pdf]

```
                                   1         .         21  1.30    .         41        .  1.61 2.34
4AMJ           MG[                            ]AELLSQQWEAGMSLLMALVVLLIVAGNVLVIAAIGsTQR
β1AR           MGDGWLPPDCGPHNRSGGGGATAAPTGSRQVSAELLSQQWEAGMSLLMALVVLLIVAGNVLVIAAIGRTQR
humanTAAR1     ------------------MMPFCHNIINISCVKNNWSNDVRASLYSLMVLIILTTLVGNLIVIVSISHFKQ
mouseTAAR3     ----------MDLIYIPEDLSSCPKFGNKSCPPTNRSFRVRMIMYLFMTGAMVITIFGNLVIIISISHFKQ
elephantTAAR7a ---------MSTELSPPAPVQLCYENLNGSCVKTSYSPGPRVMLYLVFGSGAVLAVFGNLLVMISMLHFKQ
mouseTAAR8a    ----------MTSNFSQPALQLCYENTNGSCIKTPYSPGPRVILYMVYGFGAVLAVCGNLLVVISVLHFKQ
                                                           <-----------TM1--------><---

               54   61         .         81   2.67 . 3.21  101         .         121
4AMJ           LQTLTNLFITSLACADLVvGLLVVPFGATLVVRGTWLWGSFLCElWTSLDVLCVTASIETLCVIAIDRYLA
β1AR           LQTLTNLFITSLACADLVMGLLVVPFGATLVVRGTWLWGSFLCECWTSLDVLCVTASIETLCVIAIDRYLA
humanTAAR1     LHTPTNWLIHSMATVDFLLGCLVMPYSMVRSAEHCWYFGEVFCKIHTSTDIMLSSASIFHLSFISIDRYYA
mouseTAAR3     LHSPTNFLILSMATTDFLLGFVIMPYSMVRSVESCWYFGDSFCKFHASFDMMLSLTSIFHLCSIAIDRFYA
elephantTAAR7a LHSPANFLIASLACADFLVGVTVMPFSTVRSVESCWYFGEIYCTFHSCFNGSFCYASIFHLCFISVDKFIA
mouseTAAR8a    LHSPANFLIASLASADFLVGISVMPFSMVRSIESCWYFGDAFCSLHSCCDVAFCYSSVLHLCFISVDRYIA
                                                        Δ          ΔΔ         Δ
               IC1--><----------TM2---------><------EC1-------><---------TM3---------><

               125       .  3.65 4.39 .      151     4.61        .         181   5.36 .
4AMJ           ITSPFRYQSLMTRARAKVIICTVWAISALVSFLPIMMHWWRDEDPQ-ALKCYQDPGCCDFVTNRAYAIASS
β1AR           ITSPFRYQSLMTRARAKVIICTVWAISALVSFLPIMMHWWRDEDPQ-ALKCYQDPGCCDFVTNRAYAIASS
humanTAAR1     VCDPLRYKAKMNILVICVMIFISWSVPAVFAFGMIFLELNFKGAEEIYYKHVHCRGGCSVFFSKISGVLTF
mouseTAAR3     VCDPLHYTTTMTVSMIKRLLAFCWAAPALFSFGLVLSEANVSGMQS-YEILVACFNFCALTFNKFWGTILF
elephantTAAR7a VTDPLIYPTRFTASVSGICIAFSWLLSILYSFSLLCSGANETGLEE-LVSGLSCVGGCQIAVNSNWVFVNF
mouseTAAR8a    VTDPLVYPTKFTVSVSGICISISWILPLVYSSAVFYTGISAKGIES-LVSALNCVGGCQIVINQDFVLISF
                           ▲    Δ            ▲   Δ ΔΔ                Δ        ▲ Δ ▲ Δ   ▲
               -------IC2-------><-------TM4-------><------------EC2------------><----

               196  201        .  5.65      231                                  241 6.26
4AMJ           IISFYIPLLIMIFVaLRVYREAKEQIRKIDR[                            ]ASKRKTSRVMlM
β1AR           IISFYIPLLIMIFVYLRVYREAKEQIRKIDRCEGRFYGSQEQPQPPPLPQHQPILGNGRASKRKTSRVMAM
humanTAAR1     MTSFYIPGSIMLCVYYRIYLIAKEQARLISDANQKLQIGL----------------------EMKNGISQS
mouseTAAR3     TTCFFTPGSIMVGIYGKIFIVSRRHARALSDMPANTKG------------------------AVGKNLSKK
elephantTAAR7a V-LFFIPTLVMIIVYSKIFLVAKQQARKIESLSNKTETSS----------------------DSYKDRVAK
mouseTAAR8a    L-LFFIPTLVMIILYSKIFLVAKQQAVKIETSVSGNRGESSS--------------------ESHKARVAK
               -----TM5---------><---------------------------IC3----------------------

               245       .         261       .  6.62 7.28        .         301  7.54 .
4AMJ           REHKALKTLGIIMGVFTLCWLPFFLVNIVNVFNRDLVPDWLFVaFNWLGYANSAmNPIIYCR-SPDFRKAF
β1AR           REHKALKTLGIIMGVFTLCWLPFFLVNIVNVFNRDLVPDWLFVFFNWLGYANSAFNPIIYCR-SPDFRKAF
humanTAAR1     KERKAVKTLGIVMGVFLICWCPFFICTVMDPFLHYIIPPTLNDVLIWFGYLNSTFNPMVYAFFYPWFRKAL
mouseTAAR3     KDRKAAKTLGIVMGVFLACWLPCFLAVLIDPYLDYSTPIIVLDLLVWLGYFNSTCNPLIHGFFYPWFRKAL
elephantTAAR7a RERKAAKTLGIAVIAFLISWLPYFIDIVIDAFLGFITPTYIYEILVWFAYYNSAMNPLIYAFFYPWFRKAI
mouseTAAR8a    RERKAAKTLGVTVVAFMVSWLPYTIDALVDAFMGFITPAYVYEICCWGTYYNSAMNPLIYAFFFPWFKKAI
               ------><-------TM6-------><-------EC3-------><---------TM7--------->

               316  321        .
4AMJ           KRLLaFPRKADRRLhhhhhh[
β1AR           KRLLCFPRKADRRLHAGGQPAPLPGGFISTLGSPEHSPGGTWSDCNGGTRGGSESSLEERHSKTSRSESKM
humanTAAR1     KMMLFGKIFQKDSSRCKLFLELSS-----------------------------------------------
mouseTAAR3     QFIVSGKIFRSNSDTANLFPEAH------------------------------------------------
elephantTAAR7a KLIITGKVLRENSSTTNLFSD--------------------------------------------------
mouseTAAR8a    KLILSGEILKGHSSTANLFSE--------------------------------------------------

4AMJ                                                                     ]
β1AR           EREKNILATTRFYCTFLGNGDKAVFCTVLRIVKLFEDATCTCPHTHKLKMKWRFKQHQA
humanTAAR1     -----------------------------------------------------------
mouseTAAR3     -----------------------------------------------------------
elephantTAAR7a -----------------------------------------------------------
mouseTAAR8a    -----------------------------------------------------------
```

S5 Fig. Multiple alignment of the four TAAR and the turkey β_1-adrenergic receptor proteins. Protein sequences of two primary amine detecting TAARs (human TAAR1: NP_612200 and mouse TAAR3: NP_001008429) and two tertiary amine detecting TAARs (elephant TAAR7a: XP_003404143 and TAAR8a: NP_001010830) are aligned with the sequence of the turkey β_1-adrenergic receptor (β_1AR: P07700). The position number at the top of the alignment starts at the beginning of the human TAAR1 sequence. Position numbers based on the scheme proposed by Ballesteros and Weinstein (1995) are also shown diagonally for the start and end of each transmembrane region of β_1AR. Approximate regions for transmembranes (TM1-TM7), intracellular loops (IC1-IC3), and extracellular loops (EC1-EC3) are indicated below each alignment block. The first lines of the alignment show the sequence the protein structure (4AMJ) is based on. In order to improve expression and to obtain crystals, eight thermostabilizing point mutations, a His-tag at the C-terminus, and truncations (at N-terminus, third intracellular loop, and C-terminus) were introduced (Warne *et al.*, 2012). These changes are indicated by lower cases and square brackets in the 4AMJ sequence. Residues assigned for alpha helices in 4AMJ are shown with white letters on black background. 26 residues suggested to involve with agonist binding to the β_1AR are shown with blue background (Warne *et al.*, 2011, 2012). For the β_1AR and TAAR protein sequences, residues predicted to be in transmembrane regions by Phobius (Kall *et al.*, 2007) are shown with gray background. The residues surrounding the main and minor ligand-binding pockets in the β_1AR are shown with cyan and magenta background (Nygaard *et al.*, 2009; Rosenkilde *et al.*, 2010). 29 ligand-binding sites identified by Kleinau *et al.*, (2011) are shown with green background in the human TAAR1. Among them, the residues conserved among human TAARs (including both primary amine detectors and tertiary amine detectors), adrenergic receptors, as well as other biogenic amine receptors are shown with red fonts. Those in the human TAAR1 identical or similar to the residues in the corresponding position of biogenic amine receptors are shown with yellow fonts. Positively selected sites identified by our PAML analysis are shown with triangles below the alignment: red and green are sites identified by the site and branch-site models, respectively, in TAAR7, and purple and brown are sites identified by the site and branch-site models, respectively, in TAAR8. Closed triangles indicate sites identified with posterior probabilities higher than 0.95. See S3 and S4 Tables for details.

References:

Ballesteros JA, Weinstein H. 1995. Integrated methods for the construction of three-dimensional models and computational probing of structure-function relations in G protein-coupled receptors. In: CS Stuart, editor. Receptor Molecular Biology: Academic Press. p. 366-428.

Kall L, Krogh A, Sonnhammer ELL. 2007. Advantages of combined transmembrane topology and signal peptide prediction-the Phobius web server. *Nucleic Acids Res*. 35:W429-432.

Kleinau G, Pratzka J, Nurnberg D, Gruters A, Fuhrer-Sakel D, Krude H, Kohrle J, Schoneberg T, Biebermann H. 2011. Differential modulation of Beta-adrenergic receptor signaling by trace amine-associated receptor 1 agonists. *PLoS ONE*. 6:e27073.

Nygaard R, Frimurer TM, Holst B, Rosenkilde MM, Schwartz TW. 2009. Ligand binding and micro-switches in 7TM receptor structures. *Trends Pharmacol Sci*. 30:249-259.

Rosenkilde MM, Benned-Jensen T, Frimurer TM, Schwartz TW. 2010. The minor binding pocket: a major player in 7TM receptor activation. *Trends Pharmacol Sci*. 31:567-574.

Warne T, Moukhametzianov R, Baker JG, Nehme R, Edwards PC, Leslie AGW, Schertler GFX, Tate CG. 2011. The structural basis for agonist and partial agonist action on a β_1-adrenergic receptor. *Nature*. 469:241-244.

Warne T, Edwards PC, Leslie AG, Tate CG. 2012. Crystal Structures of a Stabilized β_1-Adrenoceptor Bound to the Biased Agonists Bucindolol and Carvedilol. *Structure*. 20:841-849.
