# Supplementary material for: Molecular Evolution and Functional Divergence of Trace Amine–Associated Receptors
Source: PLoS One. 2016 Mar 10;11(3):e0151023. doi: 10.1371/journal.pone.0151023 (PMC4786312; doi:10.1371/journal.pone.0151023)
Supplement: S6 Fig — (PDF) [file pone.0151023.s006.pdf]

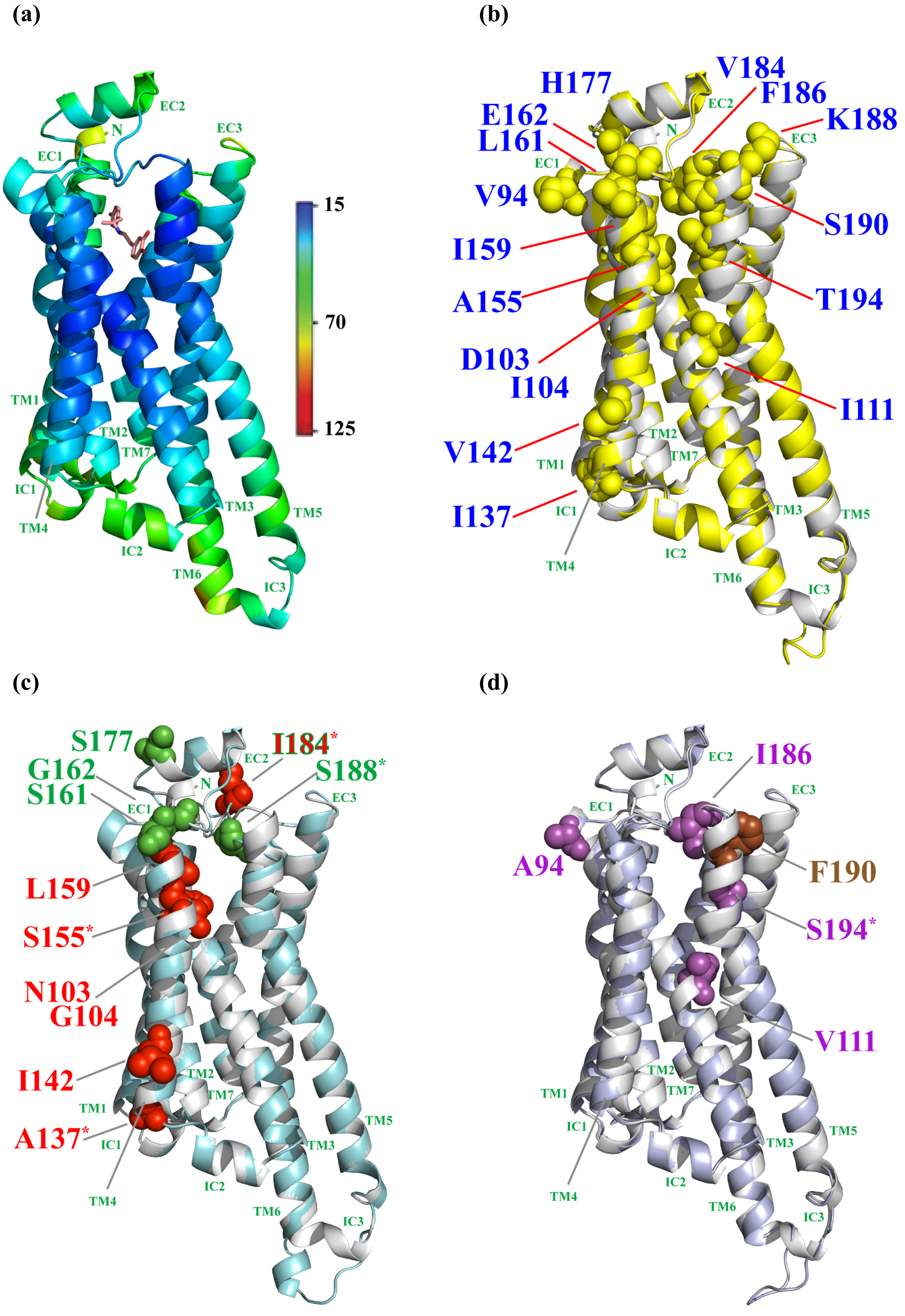
(a)
EC2
N
EC3
EC1
15
70
125
TM1
TM2
TM7
IC1
TM4
TM3
TM5
IC2
TM6
IC3
(b)
H177
V184
F186
E162
K188
L161
EC2
N
EC3
EC1
V94
I159
S190
A155
T194
D103
I104
I111
V142
TM2
TM7
TM1
I137
IC1
TM3
TM5
TM4
IC2
TM6
IC3
(c)
S177
I184*
G162
EC2
S188*
S161
N
EC1
EC3
L159
S155*
N103
G104
I142
TM2
TM1
TM7
A137*
IC1
TM5
TM3
TM4
IC2
TM6
IC3
(d)
EC2
I186
N
EC3
EC1
A94
F190
S194*
V111
TM2
TM1
TM7
IC1
TM5
TM3
TM4
IC2
TM6
IC3

S6 Fig. Modeling of the 3D-structure of TAAR proteins. The same template, the B-chain of the turkey β_1-adrenergic receptor (a: β_1AR, PDB: 4AMJ), was selected by SWISS-MODEL (http://swissmodel.expasy.org; Arnold *et al*., 2006) for modeling protein structures of the human TAAR1 (b: NP_612200), elephant TAAR7a (c: XP_003404143), and mouse TAAR8a (d: NP_001010830) (all E-values < 0.001; their sequence similarities against β_1AR, P07700, are 49.3%, 46.2%, and 43.9%, respectively). The 3D-structure of the 4AMJ (a) is color-coded based on the temperature factors (B-factors), ranging from 15.74 (blue) to 124.95 (red) (see color scale in the figure). The average B-factor is 45.52. The ligand for the β_1AR, dobutamine, is shown with the stick model. Note that the template protein contains truncations at N-terminus, third intracellular loop, and C-terminus as well as some thermostabilizing point mutations to improve expression and to obtain crystals (Warne *et al*., 2012). None of these positions were, however, overlapped with those identified to be under positive selection (see S5 Fig for more details). Predicted protein structures of the human TAAR1 (b: yellow), elephant TAAR7a (c: cyan), and mouse TAAR8a (d: light blue) are superimposed with the template structure (gray) using PyMOL. The QMEAN4 Z-scores given by SWISS-MODEL were -8.27, -8.02, and -8.37 (raw scores: 0.234, 0.250, and 0.228), respectively. The overall root-mean-square deviations (RMSDs) given by PyMOL were 0.054 Å, 0.055Å, and 0.054 Å, respectively. The N-terminal 15, 25, 23 amino acids (aa) and the C-terminal 19, 16, and 16 aa, respectively, were excluded from the modeling due to insufficient sequence similarity. Positive-selection sites identified by our PAML analysis in elephant TAAR7a (c) and mouse TAAR8a (d) are indicated by red and purple (site models) and by green and brown (branch-site models). Position 184 in elephant TAAR7a was identified by both site and branch-site models. Sites identified with higher than 0.95 posterior probabilities are indicated with asterisks. See S3 and S4 Tables for details on PAML analysis. All amino acid sites corresponding to these positive-selection sites are also mapped on human TAAR1 by yellow spheres for comparison (b). All amino acid position numbers are according to the human TAAR1 sequence. The transmembrane (TM) and internal/external loop (IC1-3 and EC1-3) regions as well as the N- terminal (N) are labeled in each structure. The C-terminal is invisible locating behind TM1. See S5 Fig for the alignment and more detailed information on these sequences.

References:

Arnold K, Bordoli L, Kopp J, Schwede T. 2006. The SWISS-MODEL workspace: a web-based environment for protein structure homology modelling. *Bioinformatics*. 22:195-201.

Warne T, Edwards PC, Leslie AG, Tate CG. 2012. Crystal Structures of a Stabilized β_1-Adrenoceptor Bound to the Biased Agonists Bucindolol and Carvedilol. *Structure*. 20:841-849.
