# Supplementary material for: Molecular Evolution and Functional Divergence of Trace Amine–Associated Receptors
Source: PLoS One. 2016 Mar 10;11(3):e0151023. doi: 10.1371/journal.pone.0151023 (PMC4786312; doi:10.1371/journal.pone.0151023)
Supplement: S7 Fig — (PDF) [file pone.0151023.s007.pdf]

(a)

	103(3.32)	104(3.33)	**137**(4.39)	142(4.44)	**155**(4.57)	159(4.61)	161	162	177	**184**	**188**(5.36)
human 1	D	I	I	V	A	I	L	E	H	V	K
mouse 3	D	M	V	R	S	V	S	E	A	L	K
mouse 7a	E	G	A	K	S	L	T	G	T	I	Q
mouse 7b	D	V	A	K	G	L	T	G	T	L	Q
mouse 7d	E	G	A	K	S	L	T	G	T	I	Q
mouse 7e	D	V	A	K	G	I	T	G	T	L	Q
mouse 7f	D	V	A	K	G	I	T	G	T	I	Q
rat 7a	E	G	A	K	S	L	T	G	T	I	Q
rat 7b	D	G	A	K	S	F	T	G	T	I	Q
rat 7c	E	G	A	K	S	L	T	G	T	V	Q
rat 7d	D	V	A	K	G	I	T	G	T	I	Q
rat 7e	D	V	A	K	S	L	T	G	T	L	Q
rat 7g	D	I	A	K	G	I	T	G	T	V	Q
rat 7h	D	M	A	K	G	I	T	G	S	I	Q
cow 7g	D	V	V	M	S	L	T	G	T	I	Q
cow 7f	E	G	V	M	S	F	T	G	T	I	Q
cow 7e	D	G	M	M	S	L	S	G	S	F	Q
cow 7d	D	G	E	M	S	L	T	G	T	I	Q
cow 7c	E	G	V	M	T	L	T	G	T	I	Q
cow 7b	E	G	V	M	T	L	T	G	T	I	Q
cow 7k	D	G	M	M	S	L	T	G	T	V	Q
horse 7b	E	G	A	M	S	L	T	G	T	I	Q
flying fox 7o	D	G	I	I	S	L	T	A	T	I	Q
flying fox 7n	D	G	I	T	Y	F	T	G	T	V	Q
flying fox 7m	D	G	I	M	N	F	T	G	T	G	Q
flying fox 7l	D	G	T	I	S	Y	T	G	T	A	Q
flying fox 7k	D	G	I	M	N	L	T	G	T	G	Q
flying fox 7j	D	G	I	I	S	F	T	G	T	I	Q
flying fox 7i	D	G	T	L	S	F	T	G	T	G	Q
flying fox 7h	D	G	I	M	Y	I	T	G	T	A	Q
flying fox 7q	D	G	I	M	N	L	T	G	T	V	Q
flying fox 7g	D	G	I	M	N	L	T	G	T	G	Q
flying fox 7f	D	G	I	M	S	F	T	G	T	G	Q
flying fox 7e	D	G	T	I	S	L	T	G	T	G	Q
flying fox 7d	D	G	I	I	Y	F	T	G	T	G	Q
flying fox 7c	D	G	I	I	S	L	T	**A**	T	**I**	Q
flying fox 7b	D	G	I	M	S	F	T	G	T	G	Q
flying fox 7a	D	G	I	M	N	L	T	G	T	G	Q
common shrew 7a	E	G	T	L	S	V	T	G	T	V	Q
common shrew 7b	E	G	T	L	S	V	T	G	T	I	Q
hedgehog 7b	E	G	M	V	S	L	T	G	T	I	Q
hedgehog 7a	D	G	M	M	S	F	T	G	T	I	Q
tenrec 7a	E	G	P	S	S	L	**S**	G	**S**	M	**S**
tenrec 7b	E	G	P	S	S	L	**S**	G	**S**	I	**S**
elephant 7a	N	G	A	I	S	L	**S**	G	**S**	I	**S**
elephant 7b	E	G	A	V	S	L	**S**	G	**S**	V	**S**
armadillo 7a	E	G	T	L	S	L	T	G	T	V	Q

(b)

	94	111(3.40)	186	190(5.38)	**194**(5.42)
human 1	V	I	F	S	T
mouse 3	S	I	F	W	L
human 8	K	V	V	W	D
mouse 8a	A	V	I	**F**	S
mouse 8b	A	A	V	W	D
mouse 8c	A	A	V	W	S
rat 8a	T	L	V	W	D
rat 8b	T	L	I	W	S
rat 8c	T	A	V	W	S
cow 8b	R	L	I	W	S
cow 8d	R	L	V	W	S
cow 8e	R	L	I	W	S
horse 8b	R	L	V	W	D
horse 8a	R	L	V	W	D
flying fox 8a	R	L	V	W	D
tenrec 8	Q	L	V	W	D
elephant 8a	Q	V	V	W	D
elephant 8b	Q	V	V	W	D

S7 Fig. Alignments of the positively selected sites identified in TAAR7 (a) and TAAR8 (b). The position numbers correspond to those given in S5 Fig. The residues identified by the branch-site models are shown in boldface. The amino acids are color-coded based on their physico-chemical properties using the Taylor color scheme (Taylor 1997). Color-coding is roughly as follows: red for negatively charged (D and E), blue/blueish for positively charged (R, K, and H), green/yellow green for hydrophobic (I, F, V, L, M, and A), blueish green for aromatic (W and Y), purple for large polar (N and Q), and reddish/orange for small (G, T, and S).

References:

Taylor WR. 1997. Residual colours: a proposal for aminochromography. *Protein Eng*. 10:743-746.
