# Supplementary material for: Molecular Evolution and Functional Divergence of Trace Amine–Associated Receptors
Source: PLoS One. 2016 Mar 10;11(3):e0151023. doi: 10.1371/journal.pone.0151023 (PMC4786312; doi:10.1371/journal.pone.0151023)
Supplement: S1 Table — (PDF) [file pone.0151023.s008.pdf]

S1 Table. The animal genomes used in this study.

Group/species	Order	Sources[a]	Coverage or version	Number of OR genes[b]	Number of TAAR genes[b]
**[Euarchontoglires]**					
Homo sapiens	Primate	NCBI (BUILD.37.2)	-	388 (414)[c]	6 (3)
Mus musculus	Rodentia	NCBI (BUILD.38.1)	-	1063 (328)[c]	15 (1)
Rattus norvegicus	Rodentia	NCBI (BUILD.4.1)	-	1259 (508)[c]	17 (2)
**[Laurasiatheria]**					
Bos taurus	Cetartiodactyla	BC	7.1×	970 (1159)[c]	21 (8)
Tursiops truncatus	Cetacea	BI	2.59×	26[c]	0 (3)
Equus caballus	Perissodactyla	BI	6.79×	NA	11 (4)
Canis familiaris	Carnivora	BI	7.6×	822 (278)[c]	2 (2)
Pteropus vampyrus	Chiroptera	BI	2.63×	672[d]	26 (10)
Myotis lucifugus	Chiroptera	BI	1.84×	659[d]	6 (1)
Sorex araneus	Insectivora	BI	1.92×	NA	9 [1] (3)
Erinaceus europaeus	Insectivora	BI	1.86×	NA	6 [2] (4)
**[Afrotheria]**					
Echinops telfairi	Afrosoricida	BI	1.90×	NA	9 [1] (7)
Loxodonta africana	Proboscidea	BI	1.94×	NA	9 [3] (3)
**[Xenarthra]**					
Dasypus novemcinctus	Cingulata	WU	2.11×	NA	5 (4)
**[Marsupialia]**					
Macropus eugenii	Diprotodontia	Ens	2.0×	NA	18 [1] (3)
Monodelphis domestica	Didelphimorphia	BI	6.8×	1198 (294)[c]	22 (4)
**[Prototheria]**					
Ornithorhynchus anatinus	Monotremata	WU	6.0×	348 (370)[c]	4 (1)
**[Sauropsida]**					
Gallus gallus	Galliformes	WU	6.6×	211 [89] (133)[e]	4 (1)
Taeniopygia guttata	Passeriformes	WU	6.3×	NA	1 (0)
Anolis carolinensis	Squamata	BI	6.3×	112 [4] (30)[e]	3 (0)
**[Amphibia]**					
Xenopus tropicalis	Anura	JGI	7.65×	824 [200] (614)[e]	7 (0)
**[Teleostei]**					
Takifugu rubripes	Tetraodontiformes	IMC	8.7×	47 [39] (39)[e]	18 (1)
Tetraodon nigroviridis	Tetraodontiformes	Gen	8.2×	11 [4] (19)[e]	34 (3)
Danio rerio	Cypriniformes	-	-	154 [1] (21)[e]	110 (10)[g]
**[Chondrichthyes]**					
Callorhinchus milii	Chimaeriformes	IMC	1.4×	1 [1] (0)[e]	2 (3)
**[Agnatha]**					
Petromyzon marinus	Petromyzontiformes	UCSC	Ver.2	32 [8] (27)[e]	25 (3)
**[Cephalochordata]**					
Branchiostoma floridae	Amphioxiformes	JGI	8.1×	31 [3] (9)[e]	0
**[Urochordata]**					
Ciona intestinalis	Enterogona	JGI	11×	0 (0)[e]	0
Ciona savignyi	Enterogona	ASL (v2.1)	-	0 (0)[e]	0
**[Cnidaria]**					
Nematostella vectensis	Actiniaria	JGI	7.8×	45[f]	0

[a]Data source abbreviations. ASL: the Arend Sidow Lab at Stanford University (http://mendel.stanford.edu/sidowlab/ciona.html), BC: Baylor College of Medicine Human Genome Sequencing Center (http://www.hgsc.bcm.tmc.edu), BI: Broad Institute at MIT (http://www.broad.mit.edu), Ens: Ensembl Genome Browser (http://www.ensembl.org), Gen: Genoscope (http://www.genoscope.cns.fr), IMC: the Institute of Molecular and Cellular Biology (http://www.imcb.a-star.edu.sg), JGI: the Joint Genome Institute (http://www.jgi.doe.gov), NCBI: National Center for Biotechnology Information (http://www.ncbi.nlm.nih.gov), WU: the Genome Sequencing Center at Washington University School of Medicine (http://genome.wustl.edu), and UCSC the University of California-San Cruz (UCSC) Genome Browser (http://genome.ucsc.edu/).

[b]Gene candidates are divided into three categories: intact, incomplete, and pseudogenes. See Table 1 for the details.

[c-g]The numbers were taken from the following literatures: Nei *et al.* (2008)[c], Hayden *et al.* (2010)[d], Niimura (2009)[e], Churcher and Taylor (2011)[f], and Hashiguchi and Nishida (2007)[g].

NA: not available.

References:

Churcher AM, Taylor JS. 2011. The Antiquity of Chordate Odorant Receptors Is Revealed by the Discovery of Orthologs in the Cnidarian *Nematostella vectensis*. *Genome Biol Evol*. 3:36-43.

Hashiguchi Y, Nishida M. 2007. Evolution of Trace Amine-Associated Receptor (TAAR) Gene Family in Vertebrates: Lineage-specific Expansions and Degradations of a Second Class of Vertebrate Chemosensory Receptors Expressed in the Olfactory Epithelium. *Mol Biol Evol*. 24:2099–2107.

Hayden S, Bekaert M, Crider TA, Mariani S, Murphy WJ, Teeling EC. 2010. Ecological adaptation determines functional mammalian olfactory subgenomes. *Genome Res*. 20:1-9.

Nei M, Niimura Y, Nozawa M. 2008. The evolution of animal chemosensory receptor gene repertoires: roles of chance and necessity. *Nat Rev Genet*. 9:951-963.

Niimura Y. 2009. On the Origin and Evolution of Vertebrate Olfactory Receptor Genes: Comparative Genome Analysis Among 23 Chordate Species. *Genome Biol Evol*. 2009:34-44.
