# Supplementary material for: Molecular Evolution and Functional Divergence of Trace Amine–Associated Receptors
Source: PLoS One. 2016 Mar 10;11(3):e0151023. doi: 10.1371/journal.pone.0151023 (PMC4786312; doi:10.1371/journal.pone.0151023)
Supplement: S3 Table — (PDF) [file pone.0151023.s010.pdf]

S3 Table. The results of PAML site-model analysis for TAAR subfamilies.

TAAR subfamily[a]	ω (M0)	2Δ*ln*L[b]		Positively selected sites[c]
		M2a–M1a	M8–M7	
TAAR1 (14)	0.1807	0 (1)	0.00038 (0.9998)	
TAAR2 (15)	0.0783	0 (1)	0.00408 (0.9980)	
TAAR3 (13)	0.0774	0 (1)	0.00532 (0.9973)	
TAAR4 (15)	0.1406	0 (1)	0.12241 (0.9406)	
TAAR5 (14)	0.1388	0 (1)	3.33783 (0.1885)	
TAAR6 (14)	0.1891	0.2721 (0.8728)	2.1408 (0.3429)	
TAAR7 (45)	0.3512	**28.3281 (<0.0001)**	**36.6892 (<0.0001)**	$103^{3.32}$ (0.69), $104^{3.33}$ (0.74), **$137^{4.39}$ (0.97)**, $142^{4.44}$ (0.89), **$155^{4.57}$ (1.00)**, $159^{4.61}$ (0.85), **184 (0.99)**
TAAR8 (16)	0.2698	0 (1)	**6.84249 (0.03267)**	94 (0.59), $111^{3.40}$ (0.78), 186 (0.62), **$194^{5.42}$ (0.95)**
TAAR9 (17)	0.1479	0 (1)	0.00024 (0.9999)	
TAAR E1 (6)	0.2835	0 (1)	0.00001 (1)	
TAAR M1 (2)	0.2444	0.0171 (0.9915)	0.06897 (0.9661)	
TAAR M2 (11)	0.3277	1.3045 (0.5209)	5.59743 (0.06089)	
TAAR M3 (9)	0.3102	0 (1)	0.32545 (0.8498)	

[a]The number of the TAAR subfamily genes we tested is given in parentheses.

[b]Likelihood-ratio test statistics. *P*-values (shown in parentheses) are obtained based on a χ^2 distribution with d.f. = 2. Significant *P*-values (< 0.05) are shown in boldfaces.

[c]Positively selected amino acid sites using the Bayes Empirical Bayes inference with the model M8. The same sites were identified with the model M2a except for two sites (94 and 186). Posterior probabilities are given in parentheses, shown in boldfaces when $P > 0.95$. The position numbers are based on the alignment shown in supplementary S5 Fig. The numbering of the Ballesteros-Weinstein scheme (Ballesteros and Weinstein 1995) is shown in superscripts.

References:

Ballesteros JA, Weinstein H. 1995. Integrated methods for the construction of three-dimensional models and computational probing of structure-function relations in G protein-coupled receptors. In: CS Stuart, editor. Receptor Molecular Biology: Academic Press. p. 366-428.
