# Supplementary material for: Molecular Evolution and Functional Divergence of Trace Amine–Associated Receptors
Source: PLoS One. 2016 Mar 10;11(3):e0151023. doi: 10.1371/journal.pone.0151023 (PMC4786312; doi:10.1371/journal.pone.0151023)
Supplement: S4 Table — (PDF) [file pone.0151023.s011.pdf]

S4 Table. The results of PAML branch-site model analysis.[a]

TAAR subfamily[b]	Foreground branch	$2\Delta ln\mathrm{L}$[c]	Proportion of site class	ω	Positively selected sites[d]
TAAR7 (45)	flying fox TAAR7c	3.9934 (0.0457)	0: 0.68747, 1: 0.29542, 2a: 0.01197, 2b: 0.00514	ω_0=0.11593, ω_1=1, ω_2=140.19823	A162 (0.657), I184 (0.599)
TAAR7 (45)	tenrec-elephant TAAR7	**7.2427 (0.0071)**	0: 0.69211, 1: 0.29130, 2a: 0.01167, 2b: 0.00491	ω_0=0.11524, ω_1=1, ω_2=169.33093	S161 (0.581), S177 (0.522), **S188$^{5.36}$ (0.973)**
TAAR8 (16)	mouse TAAR8a	6.0053 (0.0142)	0: 0.82235, 1: 0.17302, 2a: 0.00383, 2b: 0.00081	ω_0=0.14625, ω_1=1, ω_2=777.9954	F190$^{5.38}$ (0.935)

[a]Only the results where the given foreground branch having positive selection is supported significantly are listed. These branches are indicated with red color and arrows in Fig 2.

[b]The number of the TAAR subfamily genes tested is given in parentheses.

[c]Likelihood-ratio test statistics. *P*-values (shown in parentheses) are obtained based on a χ^2 distribution with d.f. = 1. *P*-values smaller than 0.01 are shown in boldfaces.

[d]Positively selected amino acid sites using the Bayes Empirical Bayes inference. Posterior probabilities are shown in parentheses, in boldfaces when $P > 0.95$. The position numbers are based on the alignment in S5 Fig. The numbering of the Ballesteros-Weinstein scheme (Ballesteros and Weinstein 1995) is shown in superscripts.

References:

Ballesteros JA, Weinstein H. 1995. Integrated methods for the construction of three-dimensional models and computational probing of structure-function relations in G protein-coupled receptors. In: CS Stuart, editor. Receptor Molecular Biology: Academic Press. p. 366-428.
